# Supplementary figures and images for: Loss of a major venom toxin gene in a Western Diamondback rattlesnake population
Source: PLoS One. 2025 Jul 3;20(7):e0319316. doi: 10.1371/journal.pone.0319316 (PMC12225875; doi:10.1371/journal.pone.0319316)

Supplementary Figure S1 Average exclusive unique peptide counts for venom proteins

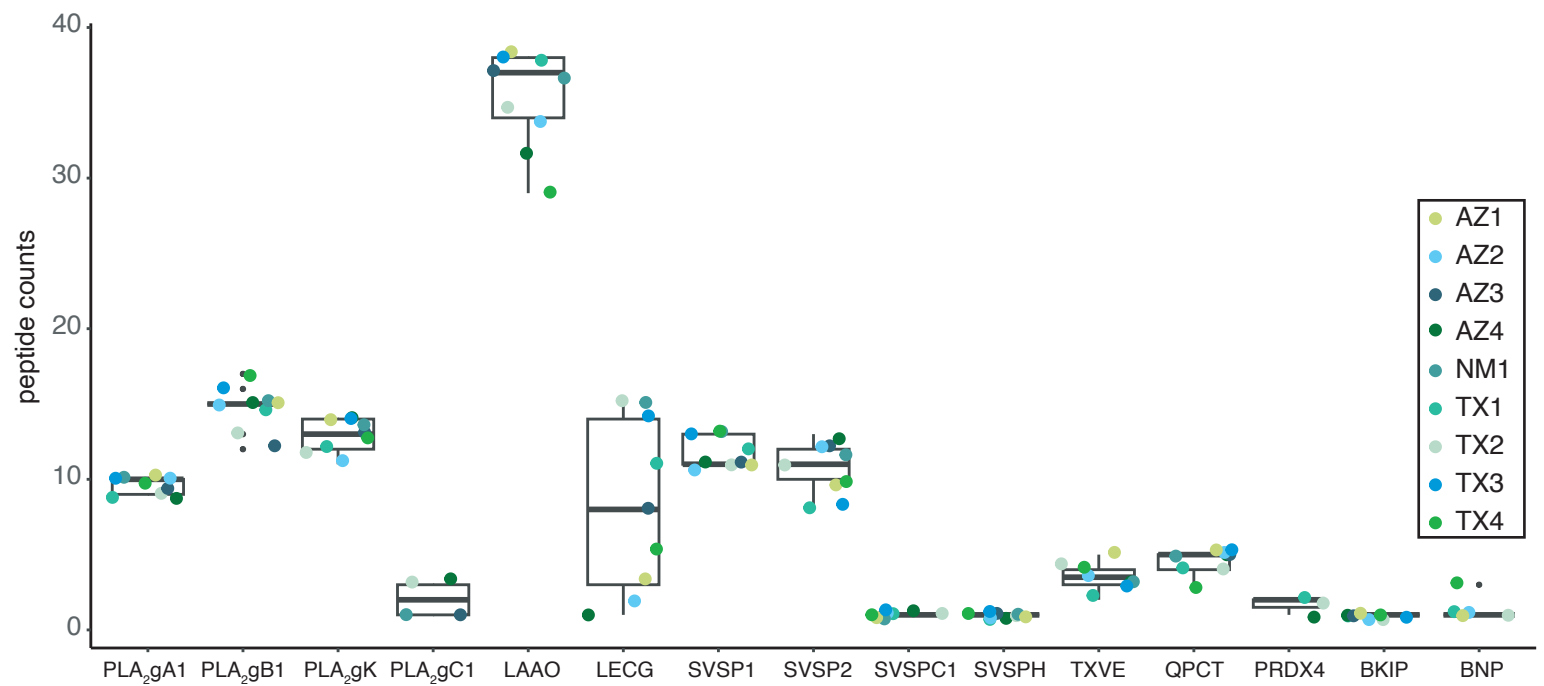

Supplement: S1 Fig — Average exclusive unique peptide counts for non-metalloproteinase venom proteins. Additional venom proteins are detected in the venom of most specimens at relatively low levels and vary little between specimens (Pla2g2g-C1, Phospholipase A2, group IIG, C1; SVSPC1, Snake venom serine proteinase-C1; SVSPH, Snake venom serine proteinase-H, TXVE, Snake venom vascular endothelial growth factor toxin; QPCT, Glutaminyl-peptide cyclotransferase; PRDX4, Peroxiredoxin-4; BKIP, Bradykinin inhibitor peptide; BNP, C-type natriuretic peptide). (PDF) [file pone.0319316.s001.pdf]

Supplementary Figure S2

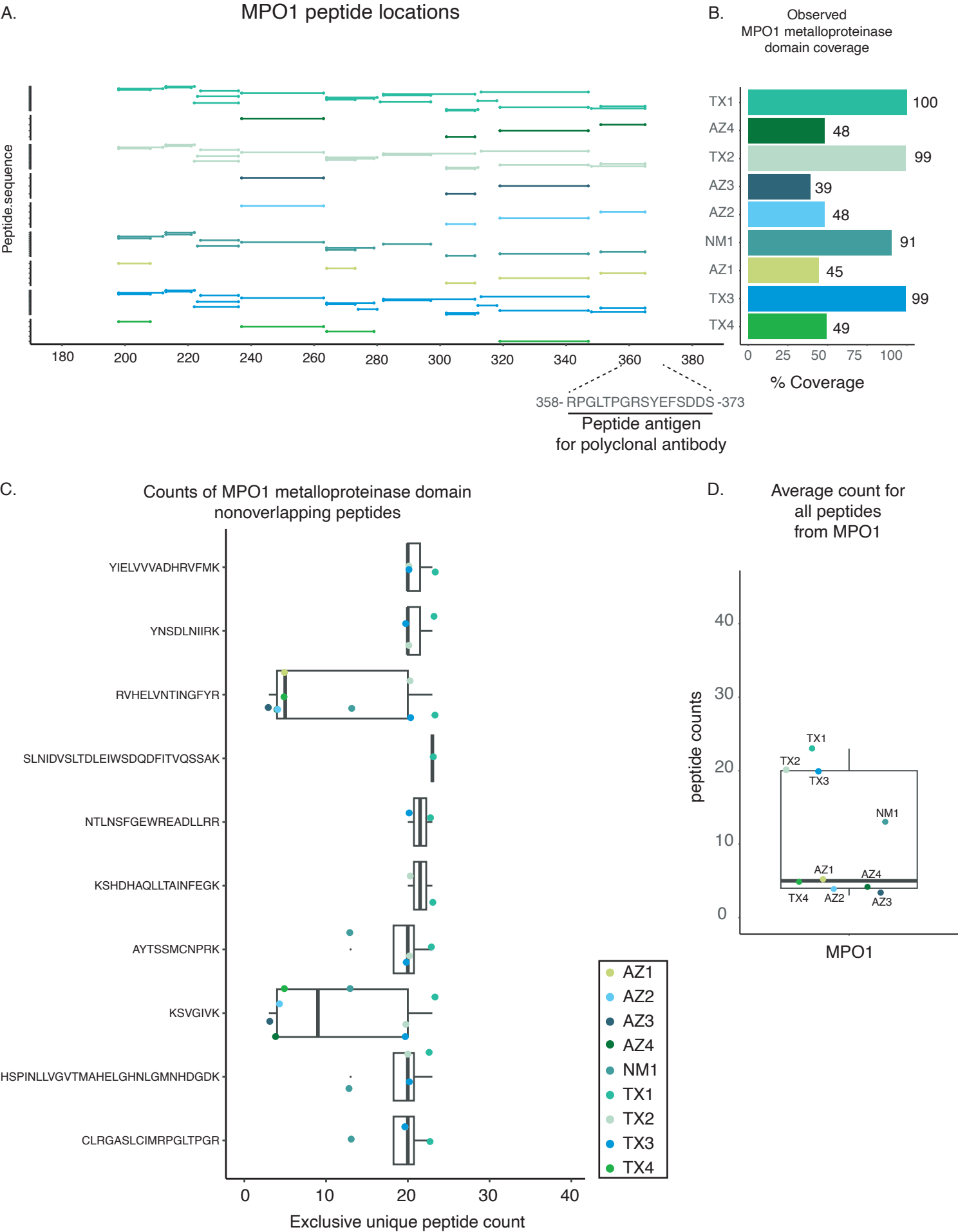

Supplement: S2–S5 Figs — These figures show the individual peptide locations (A), percentage of protein coverage (B), individual peptide counts (C) and average peptide counts of a protein (D) for MPO1 (S2 Fig), MDC8c (S3 Fig), MAD3b (S4 Fig) and MDC4 (S5 Fig). For S2-S5 Figs (A), the amino acid locations (x-axis) of exclusive unique peptides (barbell shaped line segments) are mapped to a linear representation of the respective venom protein. Each row shows peptides from a single specimen with the specimen identifier on the right side of the plot and aligned with the horizontal bar plot (B) showing observed coverage across the metalloproteinase domain. Observed coverage is the percentage of the metalloproteinase domain covered by unique peptides after removal of conserved (non-unique between paralogs) or not detected sequences. A few sequence segments of the metalloproteinases are highly conserved among MPs so peptides from those regions cannot be unambiguously assigned to single proteins and have been removed from this analysis (blank spaces shared by all specimens). The counts (x-axis) of individual non-overlapping peptides (y-axis) spanning the protein are shown for each specimen (colored dots) (C). This analysis shows that when a protein has high coverage comprised of many peptides then the associated counts of those individual peptides is often uniform and consistent with the mean count for the total protein (D). (ZIP) [file pone.0319316.s002.zip › S2Fig.pdf]

Supplementary Figure S4

A.

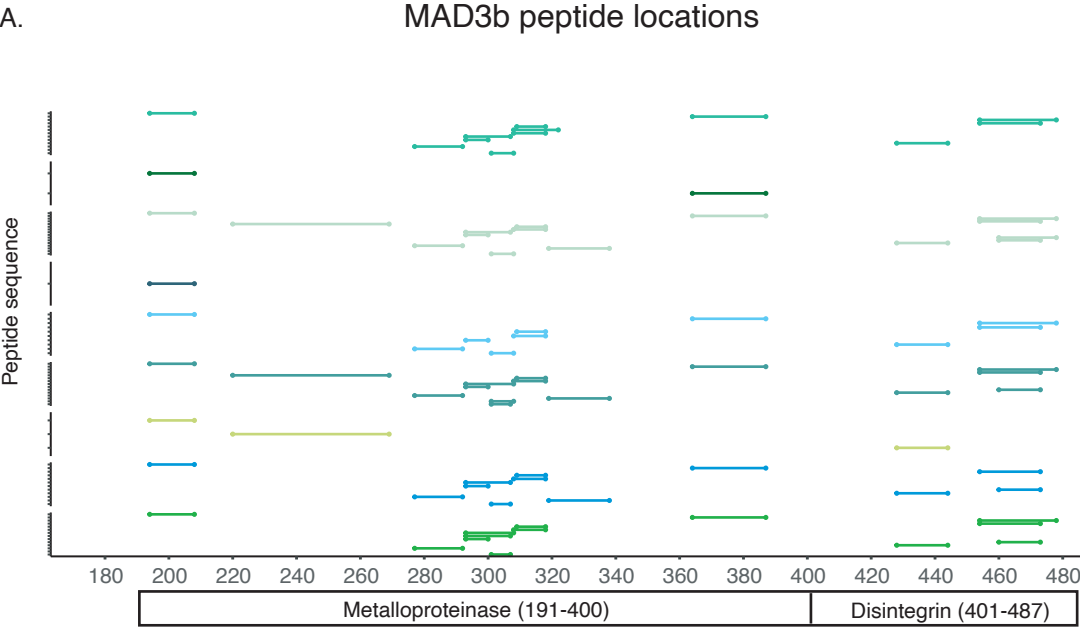

B.

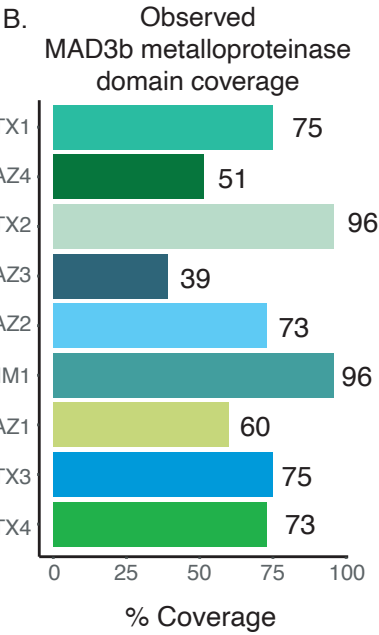

C.

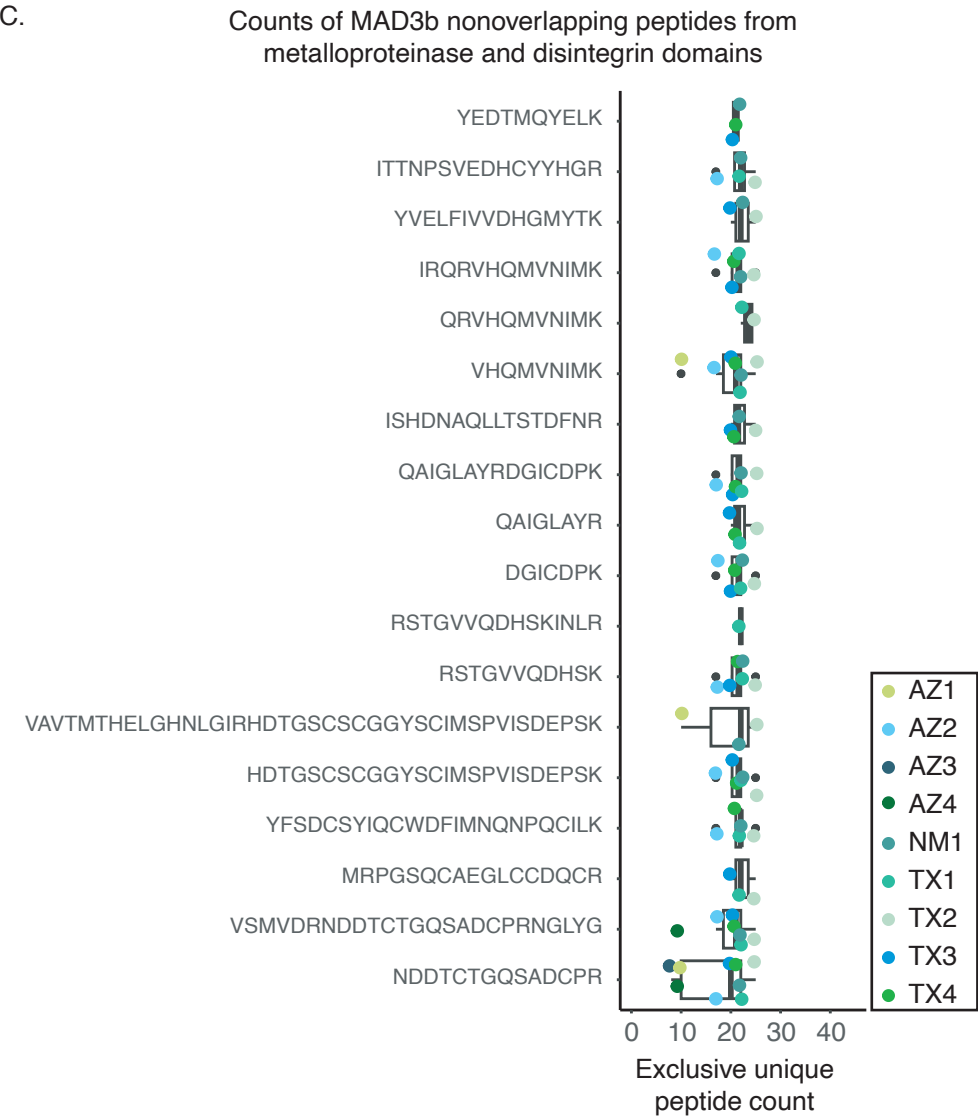

D.

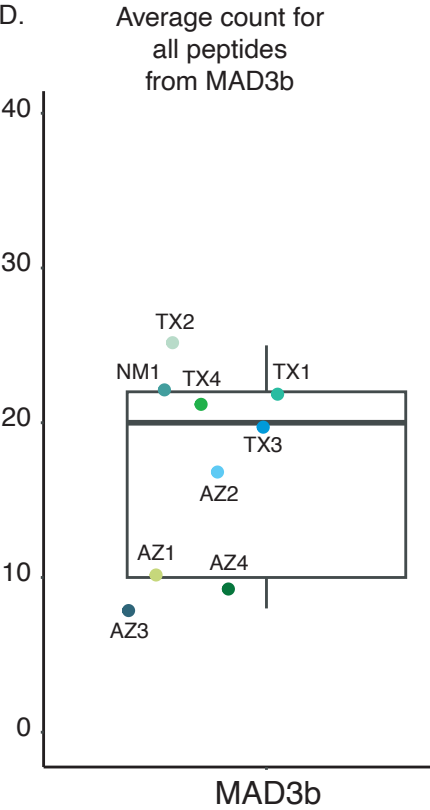

Supplement: S2–S5 Figs — These figures show the individual peptide locations (A), percentage of protein coverage (B), individual peptide counts (C) and average peptide counts of a protein (D) for MPO1 (S2 Fig), MDC8c (S3 Fig), MAD3b (S4 Fig) and MDC4 (S5 Fig). For S2-S5 Figs (A), the amino acid locations (x-axis) of exclusive unique peptides (barbell shaped line segments) are mapped to a linear representation of the respective venom protein. Each row shows peptides from a single specimen with the specimen identifier on the right side of the plot and aligned with the horizontal bar plot (B) showing observed coverage across the metalloproteinase domain. Observed coverage is the percentage of the metalloproteinase domain covered by unique peptides after removal of conserved (non-unique between paralogs) or not detected sequences. A few sequence segments of the metalloproteinases are highly conserved among MPs so peptides from those regions cannot be unambiguously assigned to single proteins and have been removed from this analysis (blank spaces shared by all specimens). The counts (x-axis) of individual non-overlapping peptides (y-axis) spanning the protein are shown for each specimen (colored dots) (C). This analysis shows that when a protein has high coverage comprised of many peptides then the associated counts of those individual peptides is often uniform and consistent with the mean count for the total protein (D). (ZIP) [file pone.0319316.s002.zip › S4Fig.pdf]

Supplementary Figure S15

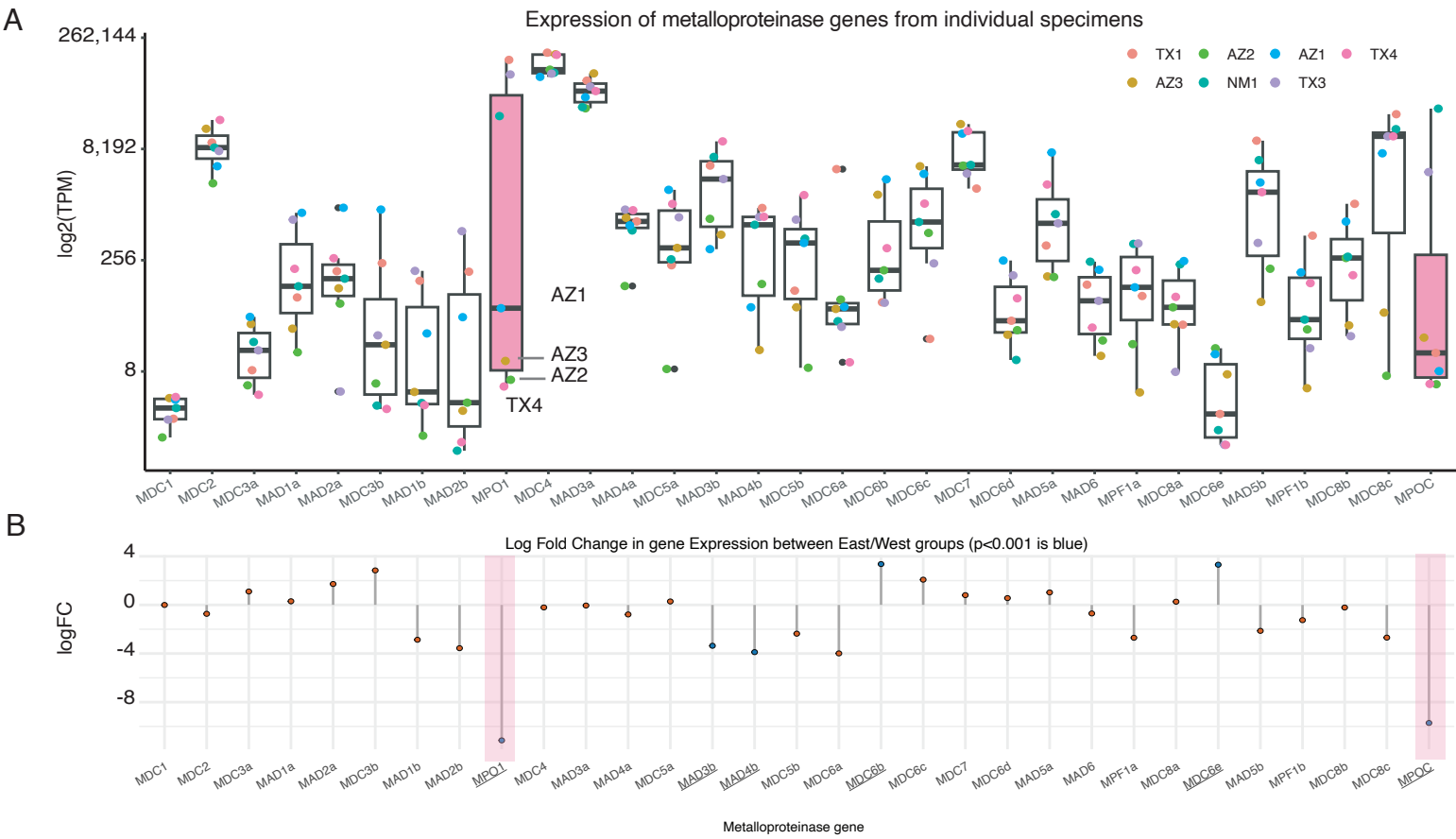

Supplement: S15 Fig — MPO1 expression correlates with geographic origin with high MPO1 expression in specimens found east of the Continental Divide (CD) and low expression in specimens found west of the CD. This motivated us to revisit our venom gland transcriptomes and perform differential gene expression analysis on east/west venom gland transcriptomes. We identified several venom metalloproteinase genes, including MPO1 (pink shading), that are differentially expressed (A, B; blue fill of ball-and-stick, P < 0.001) between the east and west groups. The x-axis shows direction of fold changes (log-transformed; logFC) as a ball-and-stick pointing upwards (higher expression in west vs east) or downwards (lower expression in the west vs east). In addition to MPO1, we also identified four additional MP genes (MAD3b, MAD4b, MDC6b and MDC6e) that are differentially expressed (B). We included MPO-C (pink shading, Atrolysin-C, a class I MP variant not present in our reference genome) in our analysis and found this gene to expressed in NM1 and TX3 (A, B). The detection of MPO-C only in high expressing MPO1 specimens suggests our approach can detect non-reference verisons of MPO1 and the identification of low-expressing specimens may not be the result of those specimens expressing a variant MPO. (PDF) [file pone.0319316.s012.pdf]

Supplementary Figure S16

A

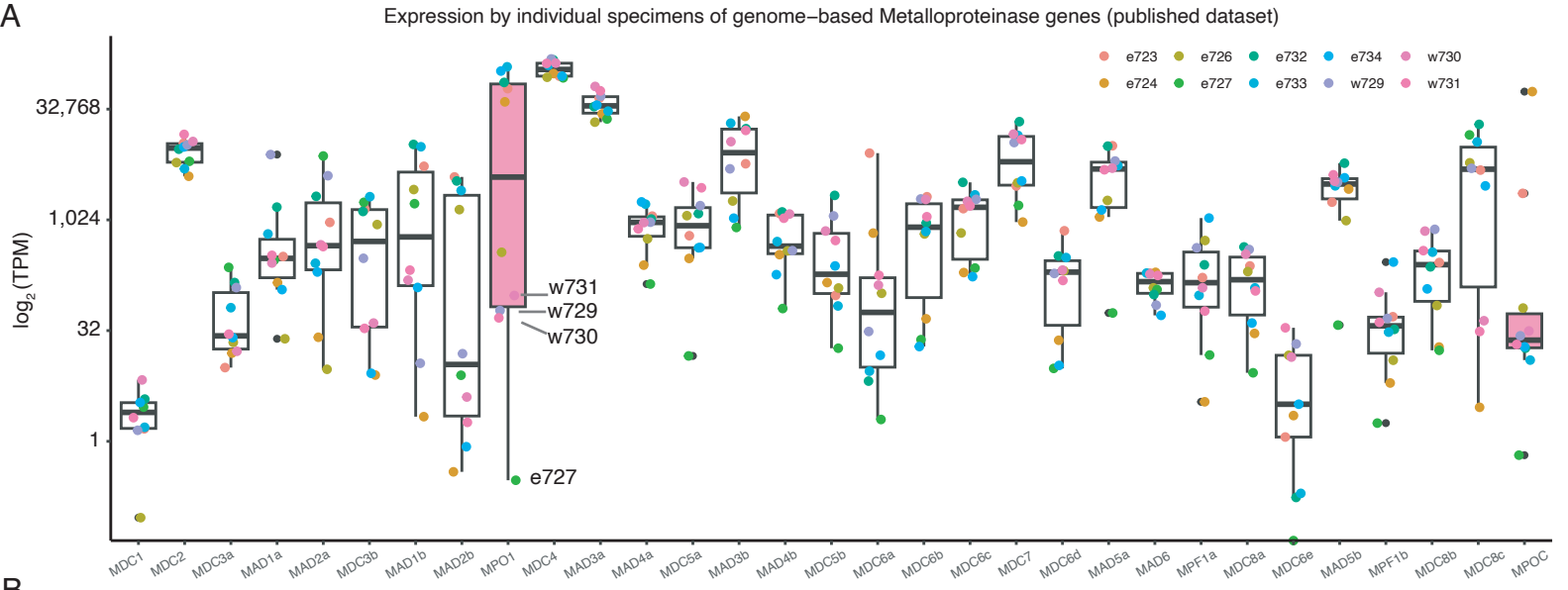

B

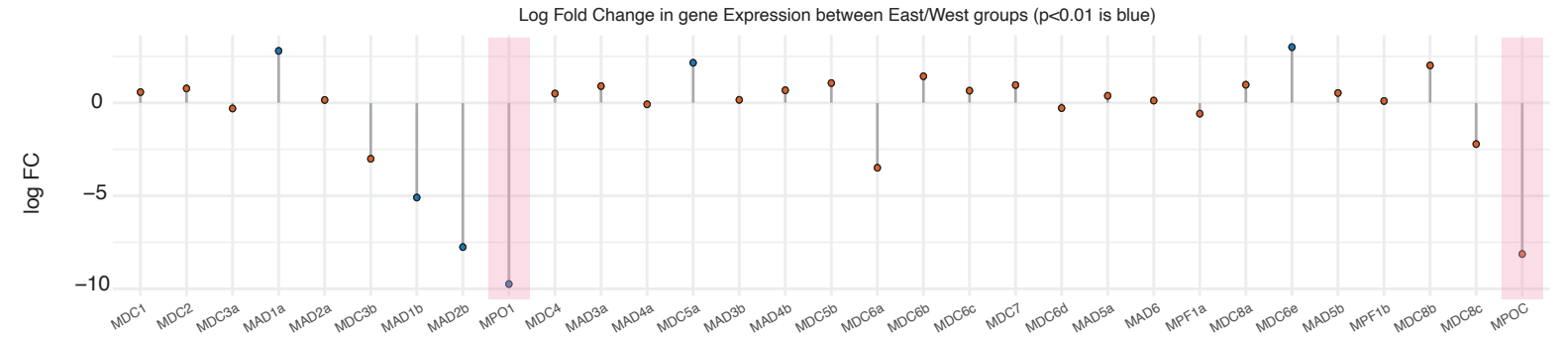

Supplement: S16 Fig — Reads from published C. atrox venom gland transcriptomes (Holding et al., 2021, PNAS) that were annotated as east or west are used with the same gene set used for the analysis in supplementary information S15 Fig to determine if any venom metalloproteinase genes are differentially expressed between the east/west groups. The variance in expression levels for all known venom metalloproteinase genes (A) for ten specimens (7, east; 3, west) generally follows the pattern shown in S15A Fig. The same DGE analysis using the published venom gland data, reveals similar decreased MPO1 expression (A, pink shading; P < 0.01). High expression of MPO-C (pink shading) was also detected in two eastern specimens. As in S15 Fig, ball-and-stick plots below the respective box plots show the log fold change in expression for specific genes between the east/west groups. Filled blue circles highlight differentially expressed genes between the east and west groups (B; P < 0.01). (PDF) [file pone.0319316.s013.pdf]

Supplementary Figure S17

Nucleic acid probe locations at three *C.atrox* genes: *MPO1*, *MDC4*, *MAD3a*

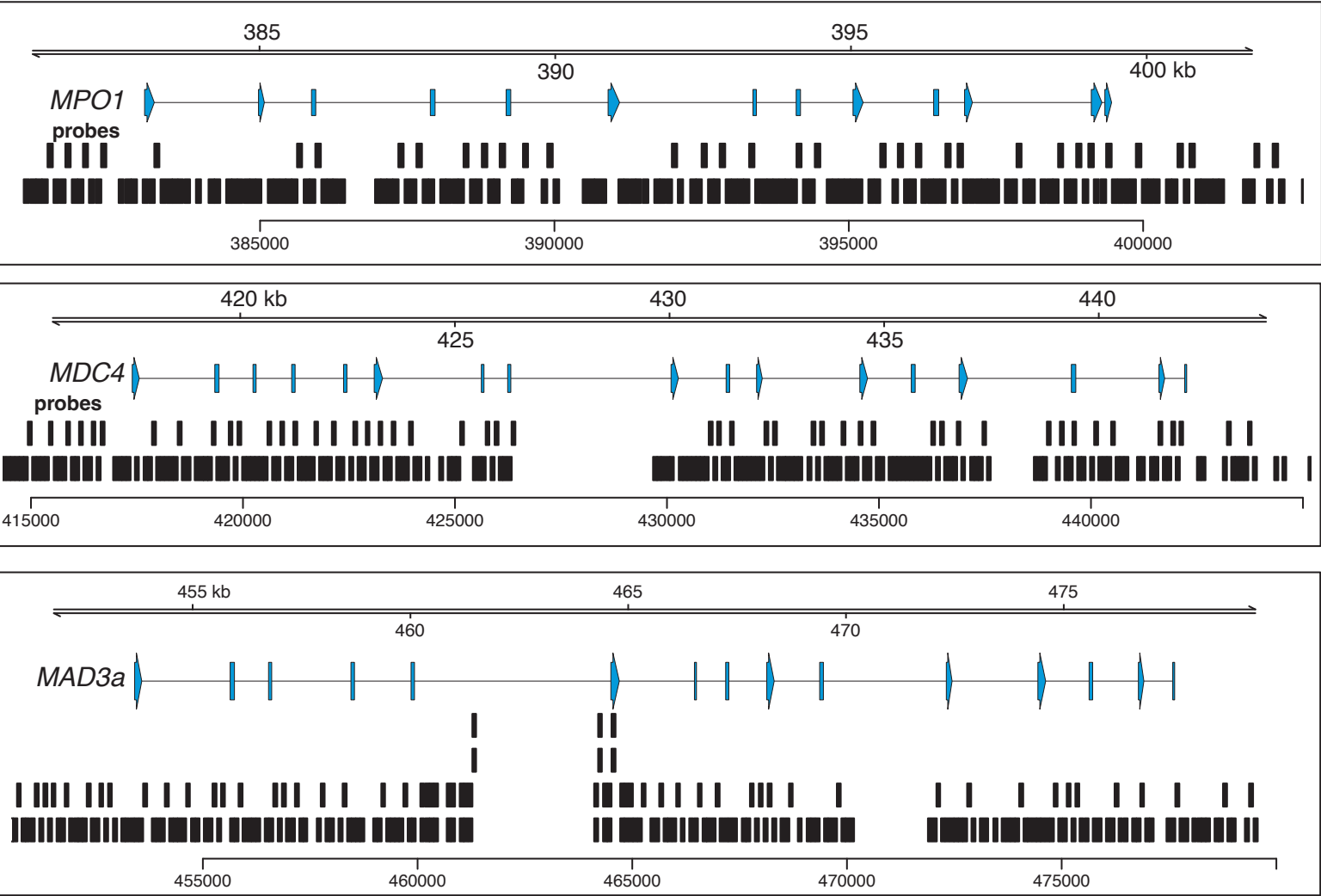

Supplement: S17 Fig — Across the entire MP gene complex probes were at an average density of one probe every 125 nucleotides but there are some larger gaps due to low sequence complexity. (PDF) [file pone.0319316.s014.pdf]

# Supplementary Figure S19

## A. *C. atrox* NM1 targeted genomic sequencing coverage at *MPO1* gene

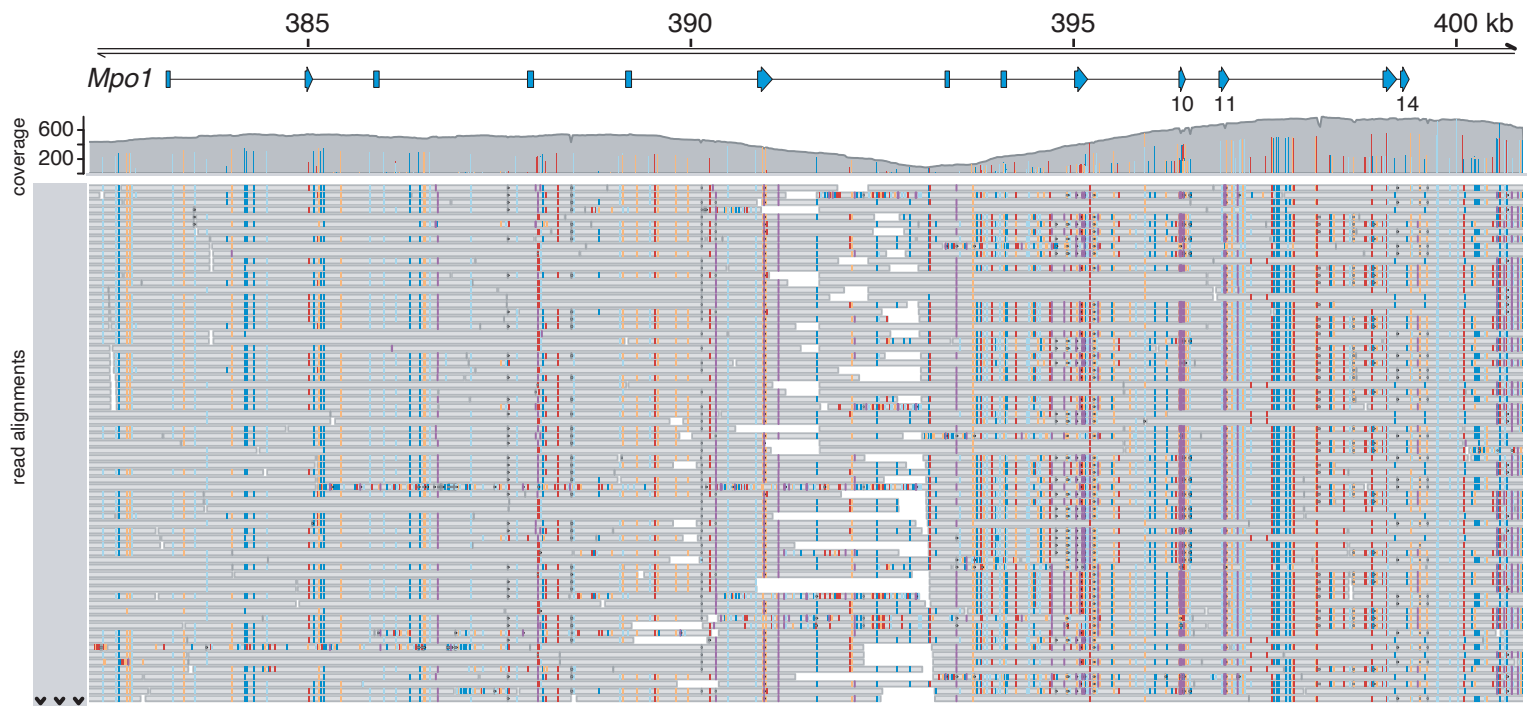

B.

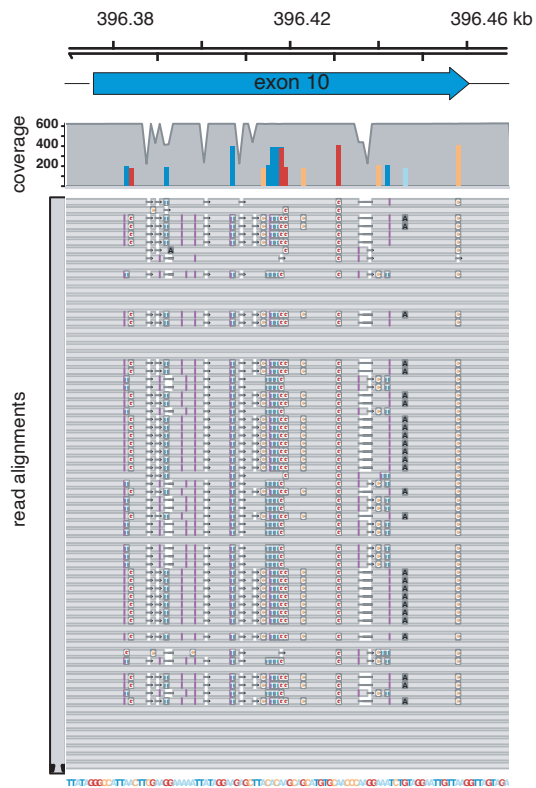

C.

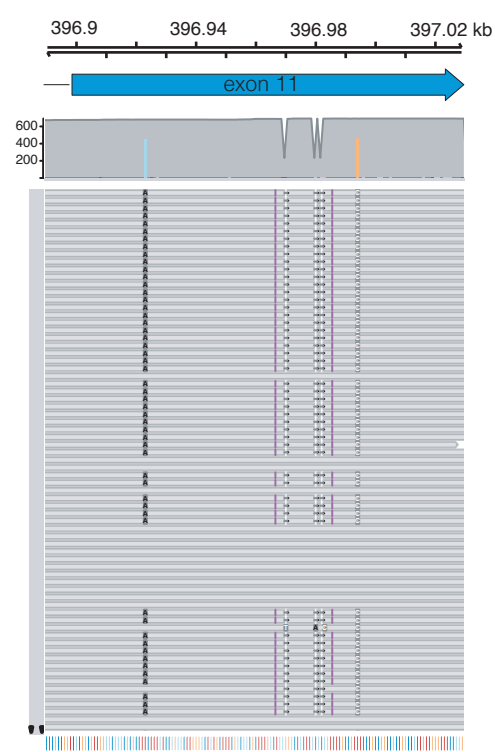

D.

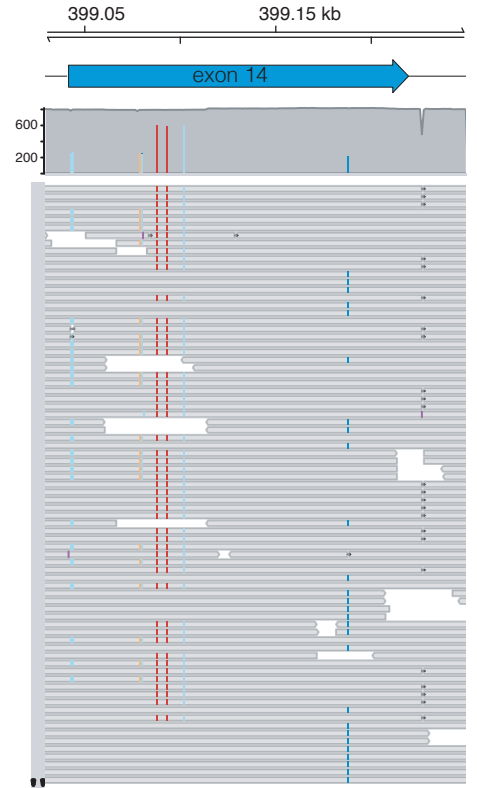

Supplement: S19 Fig — (A) Alignment of NM1 reads to the reference genome shows high coverage across the MPO1 gene with high levels of nucleotide variation. Below the coverage histogram is a pileup of the individual aligned reads (horizontal grey strips) with each nucleotide that is identical to the reference shown in grey while those that differ are shown as a colored tick mark. Zooming in on exons 10 (B), 11 (C) and 14 (D) shows the nucleotide substitutions in the coverage histogram as colored bars with the height proportional to the number of times a substitution is observed. The specific nucleotide is noted in the aligned read. Indels are shown as downward spikes in the coverage histogram and white gaps with a central horizontal bar in the aligned reads. The presence of reads that are identical to the reference sequence (grey strips) and reads that carry all of the nucleotide substitutions is evidence to the presence of two alleles at this region. In contrast, NM1 exon 14 has nucleotide variation but no indels are detected (no downward spikes in coverage and the white regions result from showing the space between the end and beginning or reads) and a hypothetical translation of exon 14 is 100% identical to the reference MPO1 sequence. (PDF) [file pone.0319316.s016.pdf]
