# Supplementary material for: Loss of a major venom toxin gene in a Western Diamondback rattlesnake population
Source: PLoS One. 2025 Jul 3;20(7):e0319316. doi: 10.1371/journal.pone.0319316 (PMC12225875; doi:10.1371/journal.pone.0319316)

Supplementary Figure S5

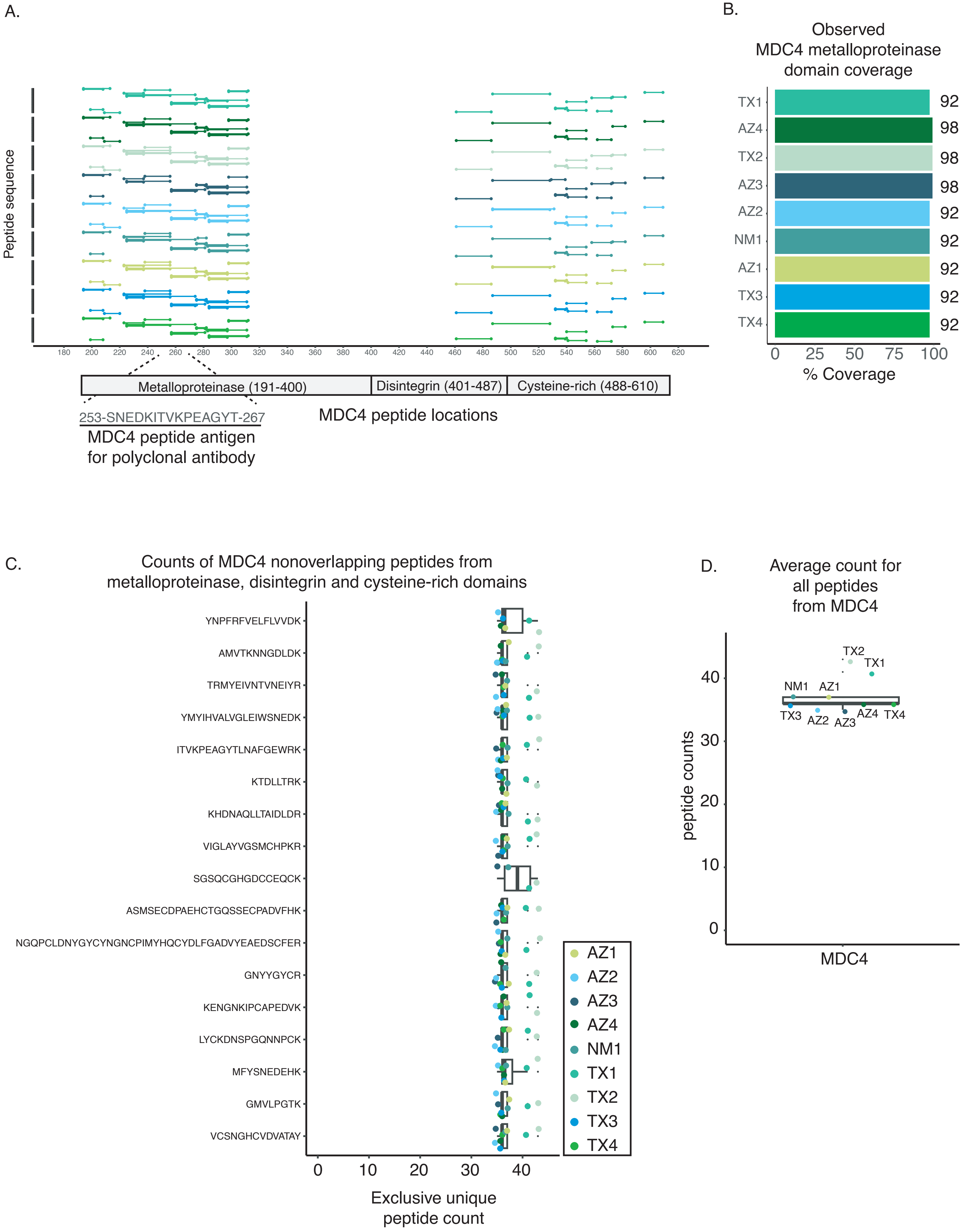

Supplement: S2–S5 Figs — These figures show the individual peptide locations (A), percentage of protein coverage (B), individual peptide counts (C) and average peptide counts of a protein (D) for MPO1 (S2 Fig), MDC8c (S3 Fig), MAD3b (S4 Fig) and MDC4 (S5 Fig). For S2-S5 Figs (A), the amino acid locations (x-axis) of exclusive unique peptides (barbell shaped line segments) are mapped to a linear representation of the respective venom protein. Each row shows peptides from a single specimen with the specimen identifier on the right side of the plot and aligned with the horizontal bar plot (B) showing observed coverage across the metalloproteinase domain. Observed coverage is the percentage of the metalloproteinase domain covered by unique peptides after removal of conserved (non-unique between paralogs) or not detected sequences. A few sequence segments of the metalloproteinases are highly conserved among MPs so peptides from those regions cannot be unambiguously assigned to single proteins and have been removed from this analysis (blank spaces shared by all specimens). The counts (x-axis) of individual non-overlapping peptides (y-axis) spanning the protein are shown for each specimen (colored dots) (C). This analysis shows that when a protein has high coverage comprised of many peptides then the associated counts of those individual peptides is often uniform and consistent with the mean count for the total protein (D). (ZIP) [file pone.0319316.s002.zip › S5Fig.pdf]
