# Supplementary material for: Loss of a major venom toxin gene in a Western Diamondback rattlesnake population
Source: PLoS One. 2025 Jul 3;20(7):e0319316. doi: 10.1371/journal.pone.0319316 (PMC12225875; doi:10.1371/journal.pone.0319316)

Supplementary Figure S6 Counts of mapped reads to reference transcript and coverage across transcript

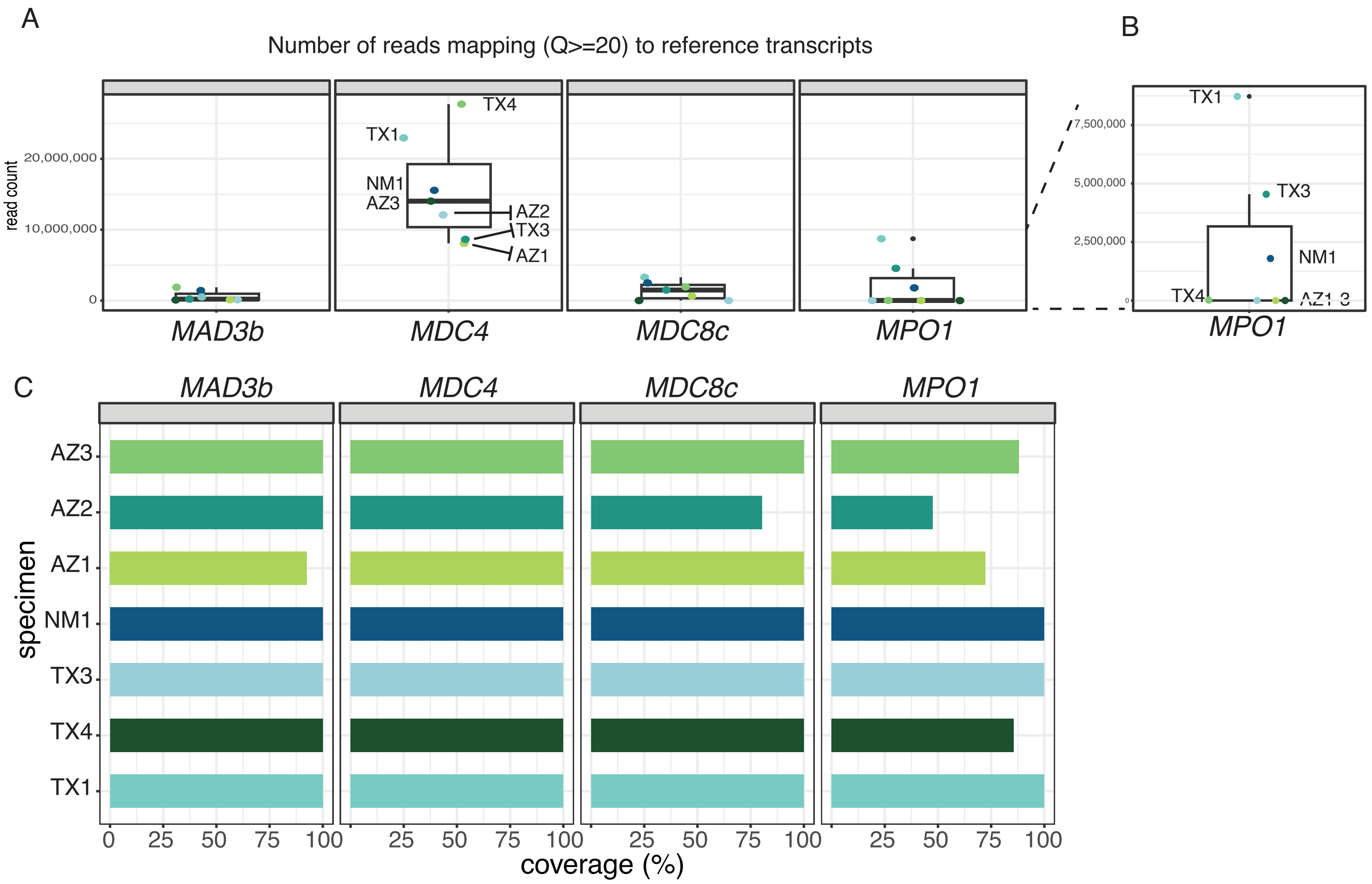

Supplement: S6 Fig — (A) Box plots showing between specimen variation in counts of mapped reads to reference transcripts. These counts are not length-normalized so longer transcripts can have higher counts and comparisons across the different classes of transcripts are not indicative of expression differences. (B) Zoomed view of the MPO1 counts highlighting the variation between specimens. (C) Horizontal bar plots showing the percent of the reference transcript covered (x-axis) by mapped reads from each specimen. (PDF) [file pone.0319316.s003.pdf]
