# Supplementary material for: Loss of a major venom toxin gene in a Western Diamondback rattlesnake population
Source: PLoS One. 2025 Jul 3;20(7):e0319316. doi: 10.1371/journal.pone.0319316 (PMC12225875; doi:10.1371/journal.pone.0319316)

Supplementary Figure S7

A. Specimens with low and zero coverage across *MPO1* reference transcript

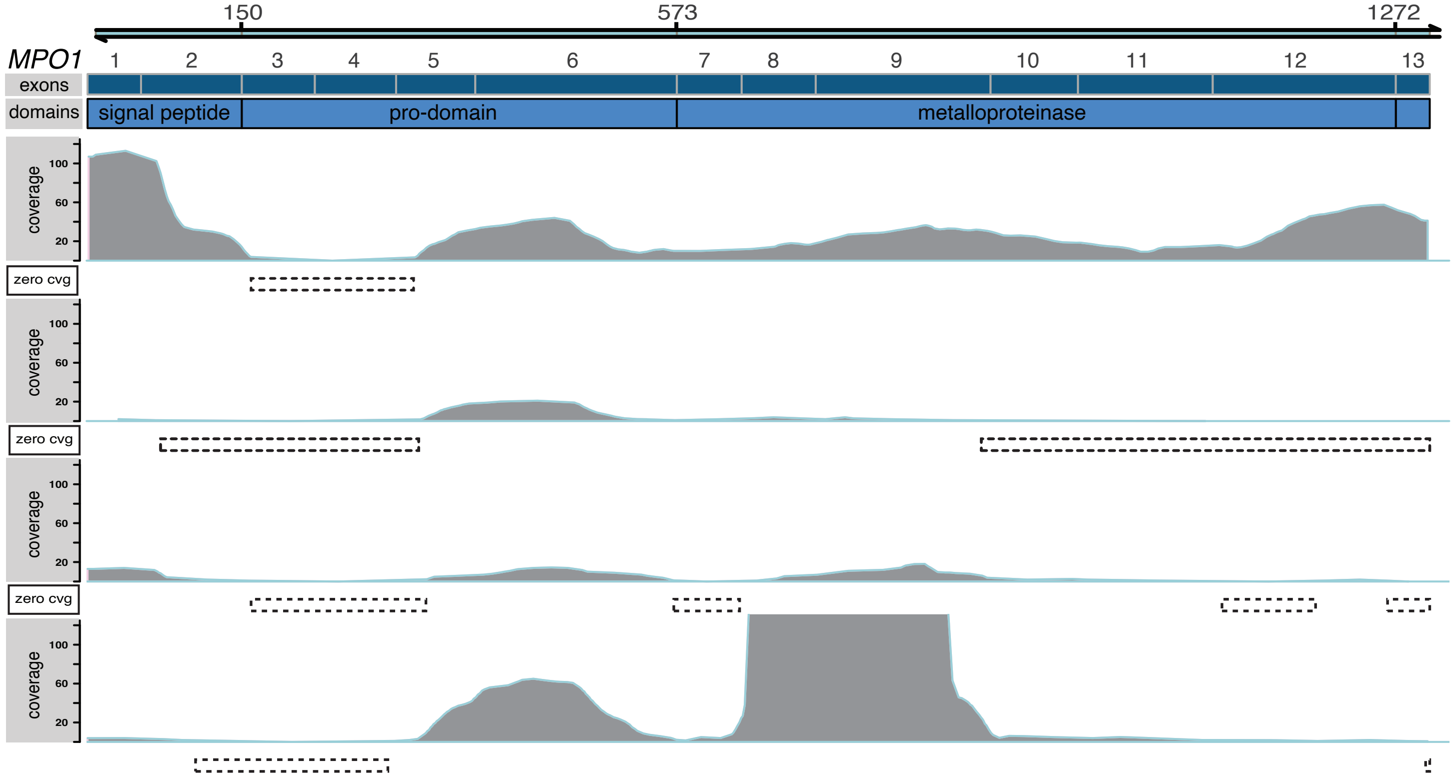

B. Specimens with high coverage across *MPO1* reference transcript

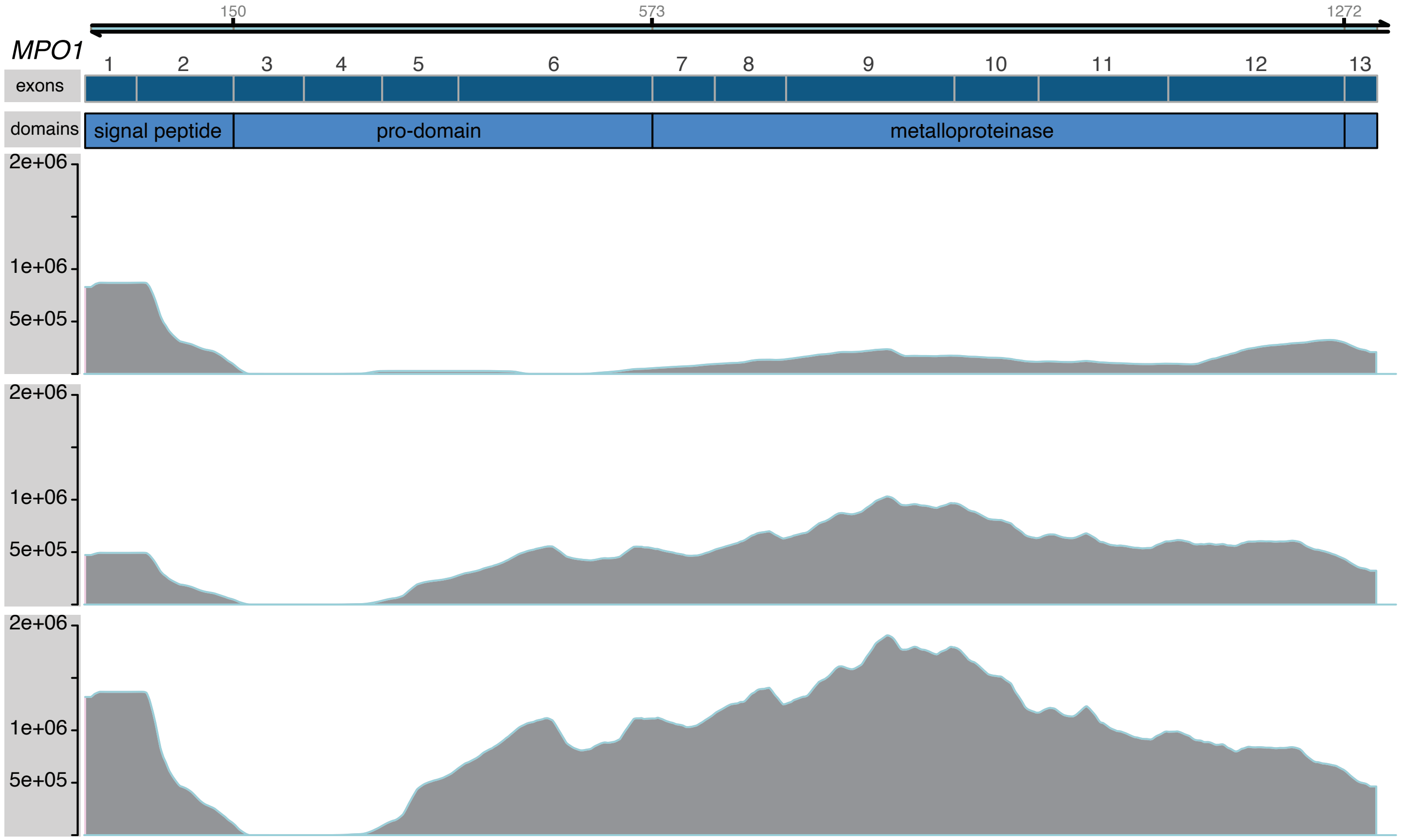

Supplement: S7 Fig — Abundant MPO1 expression correlates with complete read coverage across the MPO1 transcript. Specimens with low total read coverage (A) have gaps in coverage (dashed boxes) across the MPO1 transcript whereas specimens with high MPO1 expression (B) have complete coverage across the transcript. (PDF) [file pone.0319316.s004.pdf]
