# Supplementary material for: Loss of a major venom toxin gene in a Western Diamondback rattlesnake population
Source: PLoS One. 2025 Jul 3;20(7):e0319316. doi: 10.1371/journal.pone.0319316 (PMC12225875; doi:10.1371/journal.pone.0319316)

Supplementary Figure S8

A. Assembled transcripts aligning to the *MPO1* gene and linking multiple exons (high expressing specimens).

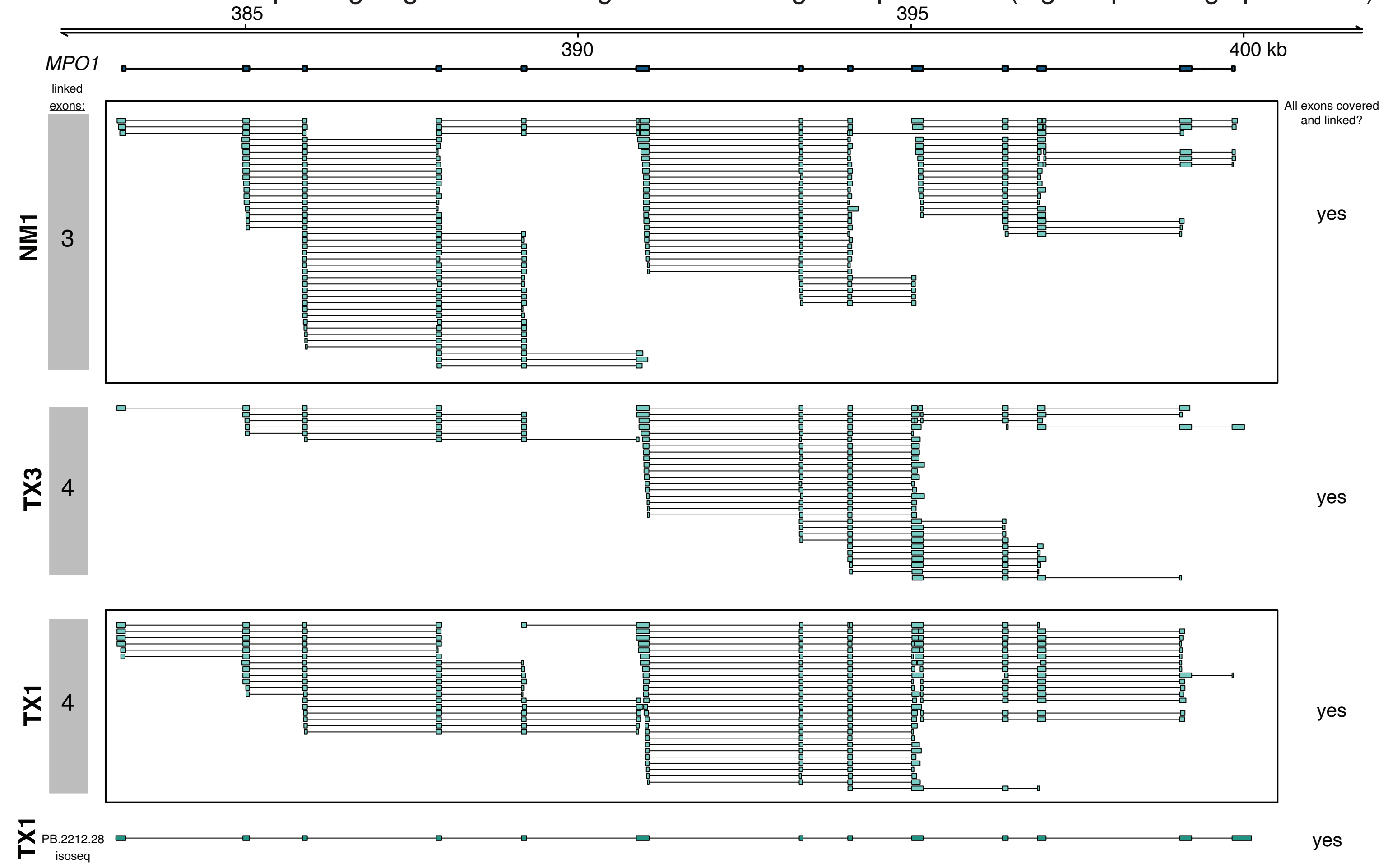

B. Assembled transcripts aligning to the *MPO1* gene and linking multiple exons (low expressing specimens).

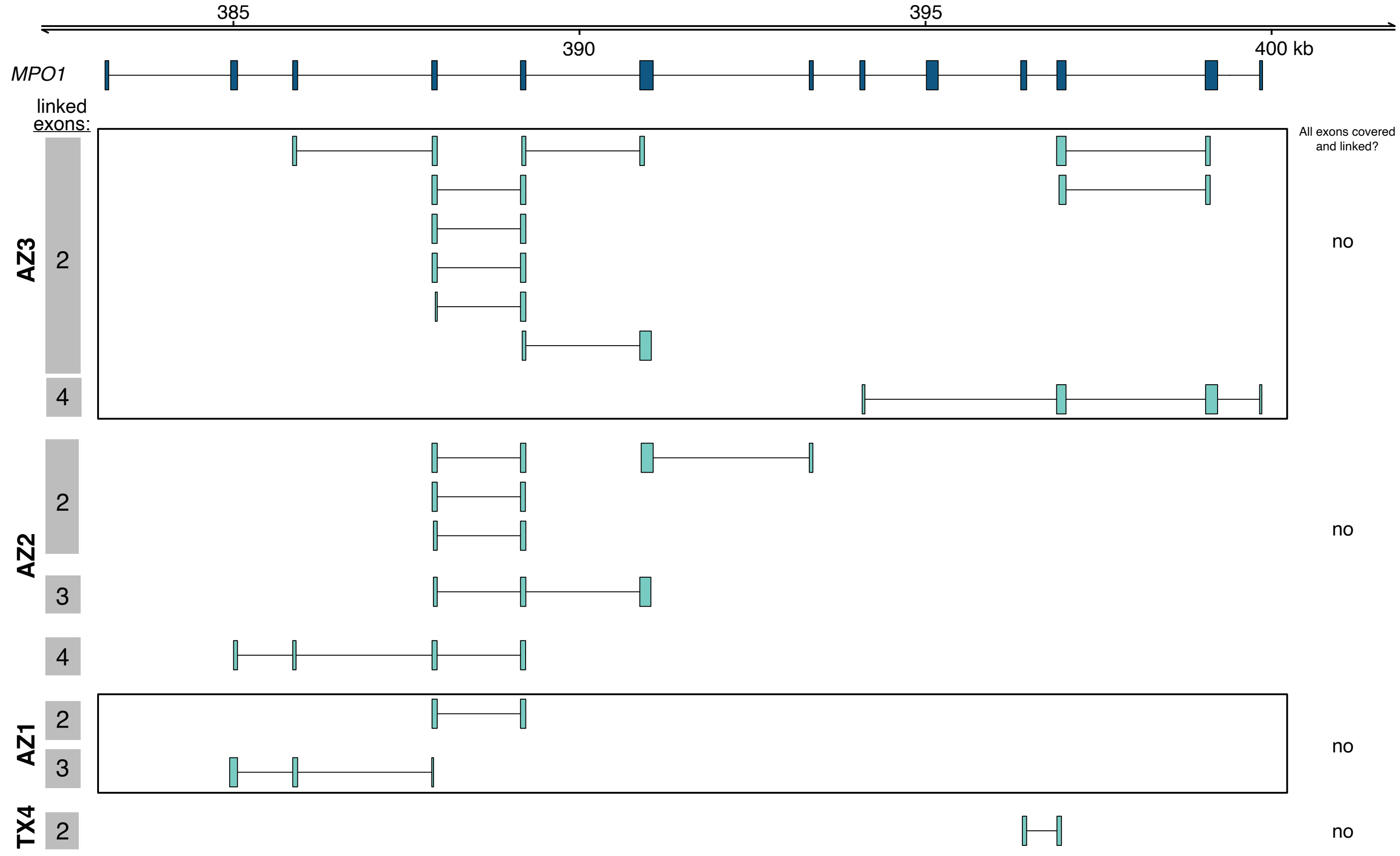

Supplement: S8 Fig — (A) Specimens with relatively high MPO1 expression have assembled transcripts that tile across the complete gene and link exons. (B) Specimens with relatively low MPO1 expression lack assembled transcripts that tile across the complete gene and link exons. Additionally, specimen NM1 has a linking transcript that connects exon 8 to exon 11 and lacks alignments to exons 9 and 10 thus suggesting the mutated exons 9 and 10 may be removed through splicing. (PDF) [file pone.0319316.s005.pdf]
