# Supplementary material for: Loss of a major venom toxin gene in a Western Diamondback rattlesnake population
Source: PLoS One. 2025 Jul 3;20(7):e0319316. doi: 10.1371/journal.pone.0319316 (PMC12225875; doi:10.1371/journal.pone.0319316)

Supplemental Figure S9

Detection of full length and novel *MPO1* isoforms with single molecule sequencing

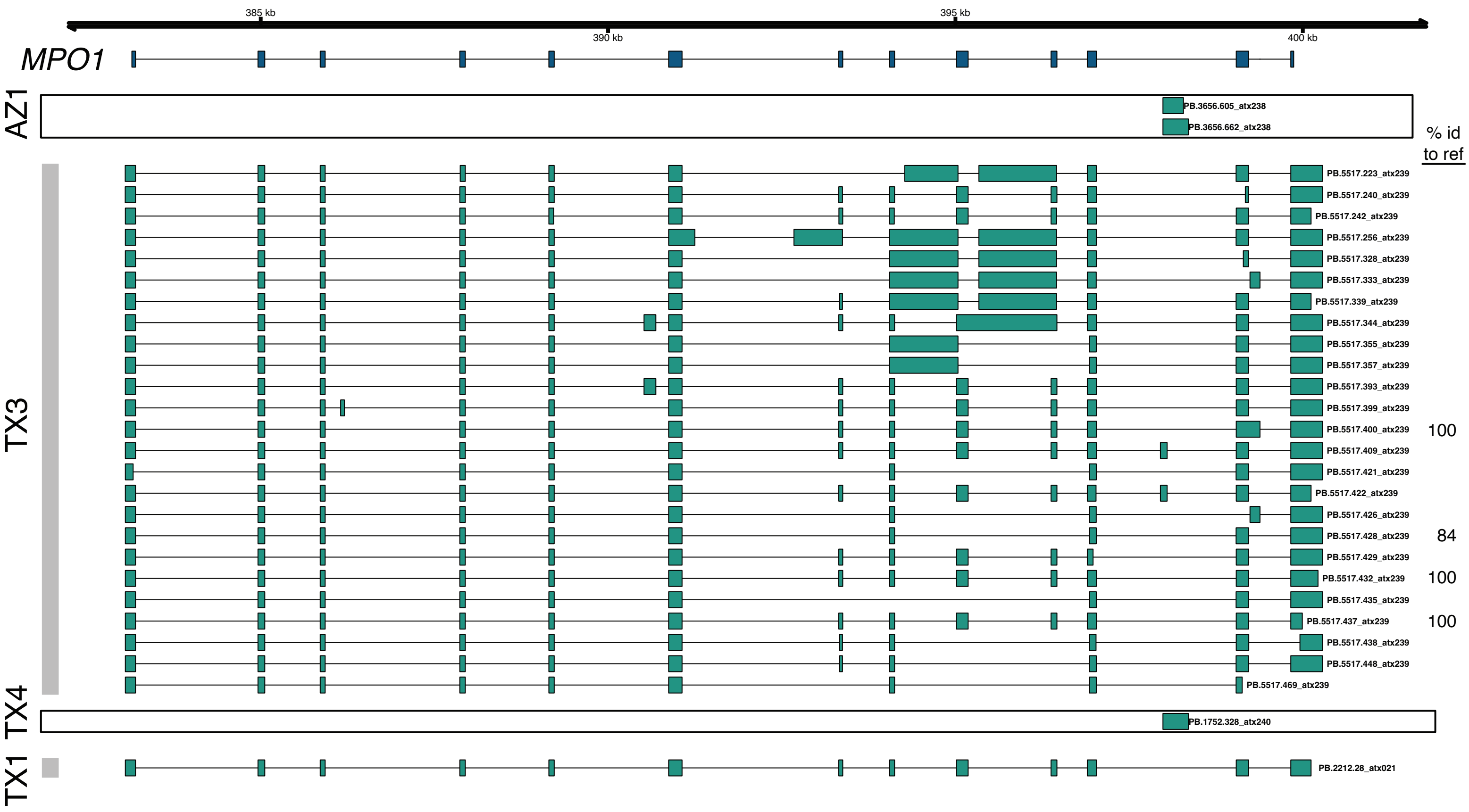

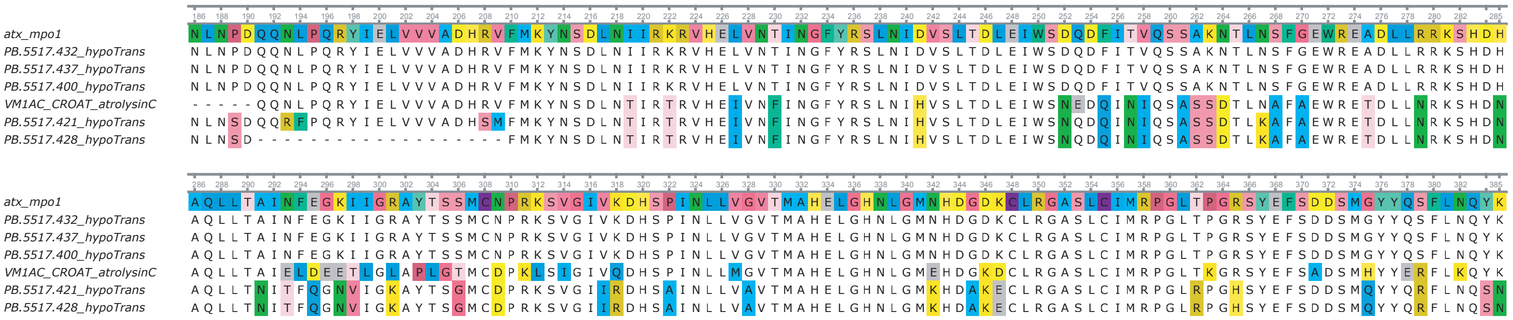

Supplement: S9 Fig — For two specimens (TX1 and TX3) a full-length MPO1 isoform is detected. For specimen TX3, novel isoforms consisting of spliced exons, novel exons and/or retained introns are also shown. For two specimens (AZ1 and TX4) with low MPO1 protein and mRNA levels no full-length MPO1 isoforms are detected. Hypothetical translations of four isoforms (PB.5517.400, PB.5517.428, PB.5517.432, PB.5517.437) and alignment to reference MPO1 sequence identified three sequences (PB.5517.400, PB.5517.432, PB.5517.437) identical to the reference sequence and one (PB.5517.428) with 18 shared amino acids (219–285) with MPO-C (atrolysin-C) but differences in the 3’ prime sequence. This shows our approach is able to detect the expression of non-reference MPO genes. (PDF) [file pone.0319316.s006.pdf]
