# Supplementary material for: Loss of a major venom toxin gene in a Western Diamondback rattlesnake population
Source: PLoS One. 2025 Jul 3;20(7):e0319316. doi: 10.1371/journal.pone.0319316 (PMC12225875; doi:10.1371/journal.pone.0319316)

Supplementary Figure S10

A. Assembled transcripts aligning to the *MDC8c* gene and linking multiple exons.

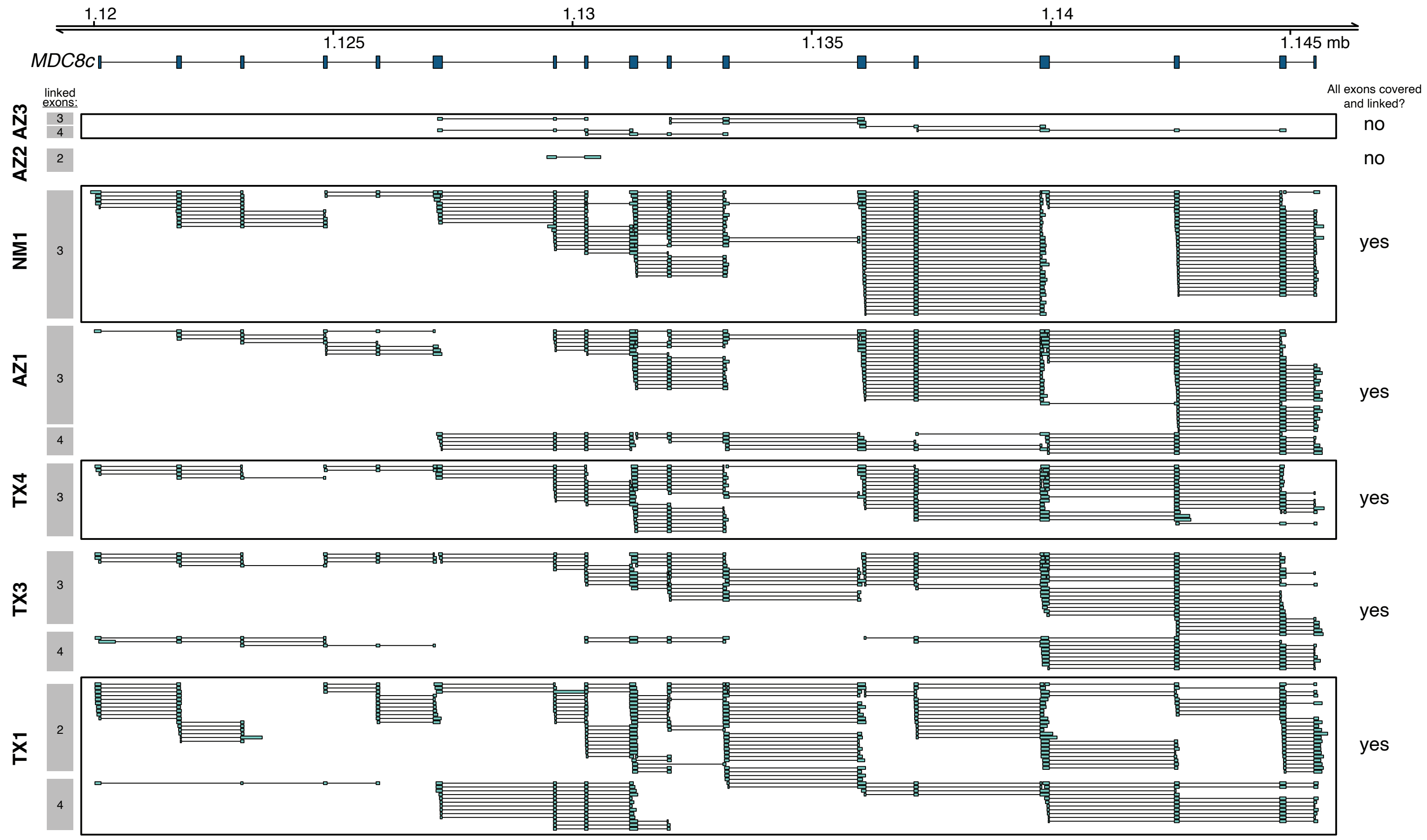

B. Full-length sequences aligning to the *MDC8c* gene.

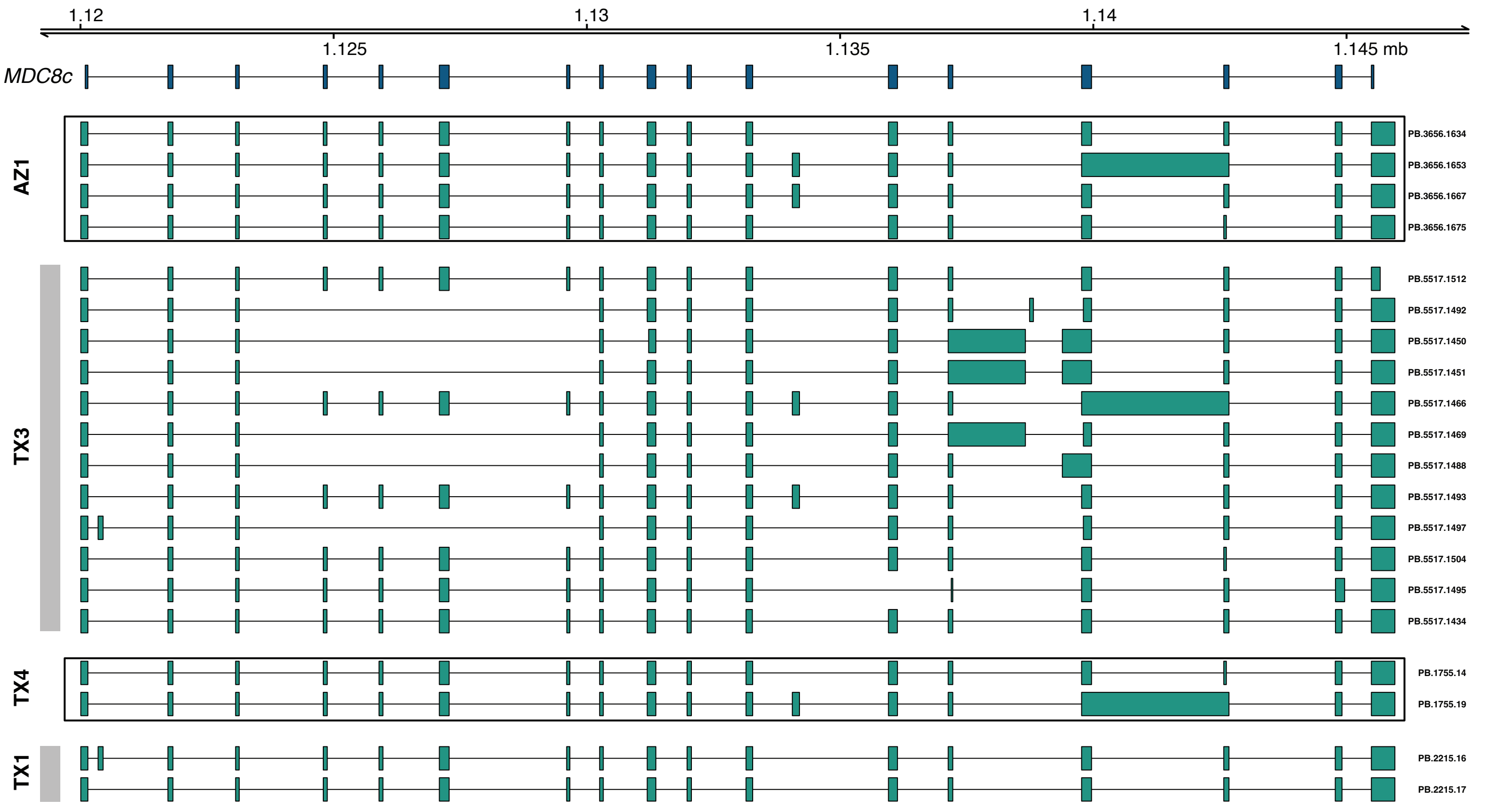

Supplement: S10 Fig — Consistency between assembled transcripts linking all exons and single molecule sequencing of full-length MDC8c isoforms. (A) MDC8c assembled transcripts that tile across the complete gene and link exons with the notable exceptions of AZ2 and AZ3. (B) Full-length MDC8c isoforms identified using single molecule sequencing. (PDF) [file pone.0319316.s007.pdf]
