# Supplementary material for: Loss of a major venom toxin gene in a Western Diamondback rattlesnake population
Source: PLoS One. 2025 Jul 3;20(7):e0319316. doi: 10.1371/journal.pone.0319316 (PMC12225875; doi:10.1371/journal.pone.0319316)

Supplementary Figure S11

A. Assembled transcripts aligning to the *MAD3b* gene and linking multiple exons.

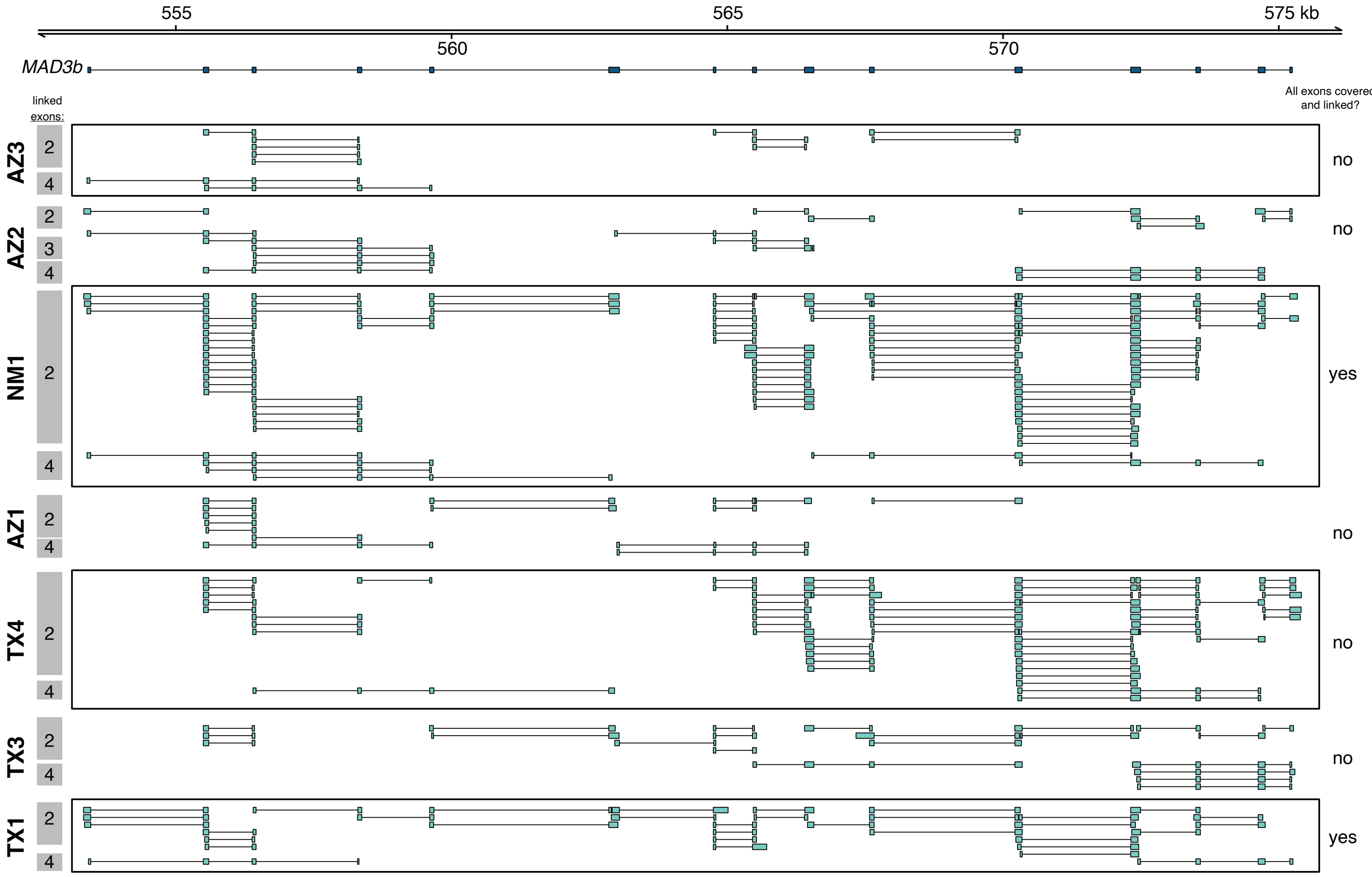

B. Full-length sequences aligning to the *MAD3b* gene.

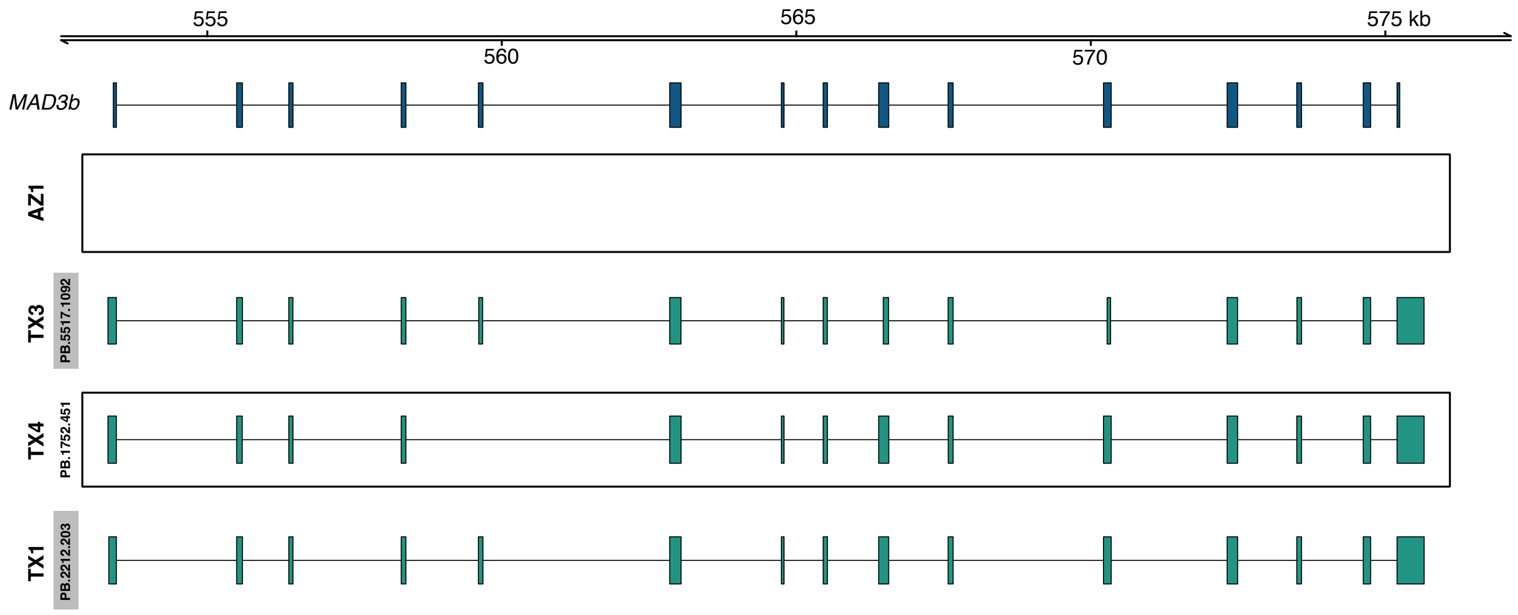

Supplement: S11 Fig — Single molecule sequencing recovers full-length MAD3b isoforms in specimens that lack assembled transcripts tiling across the gene. (A) MAD3b assembled transcripts that tile across the complete gene and link exons with the exceptions of AZ1, AZ2, AZ3, TX3, TX4. B) Full-length MAD3b isoforms identified using single molecule sequencing with the exception of AZ1. (PDF) [file pone.0319316.s008.pdf]
