# Supplementary material for: Loss of a major venom toxin gene in a Western Diamondback rattlesnake population
Source: PLoS One. 2025 Jul 3;20(7):e0319316. doi: 10.1371/journal.pone.0319316 (PMC12225875; doi:10.1371/journal.pone.0319316)

Supplementary Figure S12

A. Assembled transcripts aligning to the *MDC4* gene and linking multiple exons.

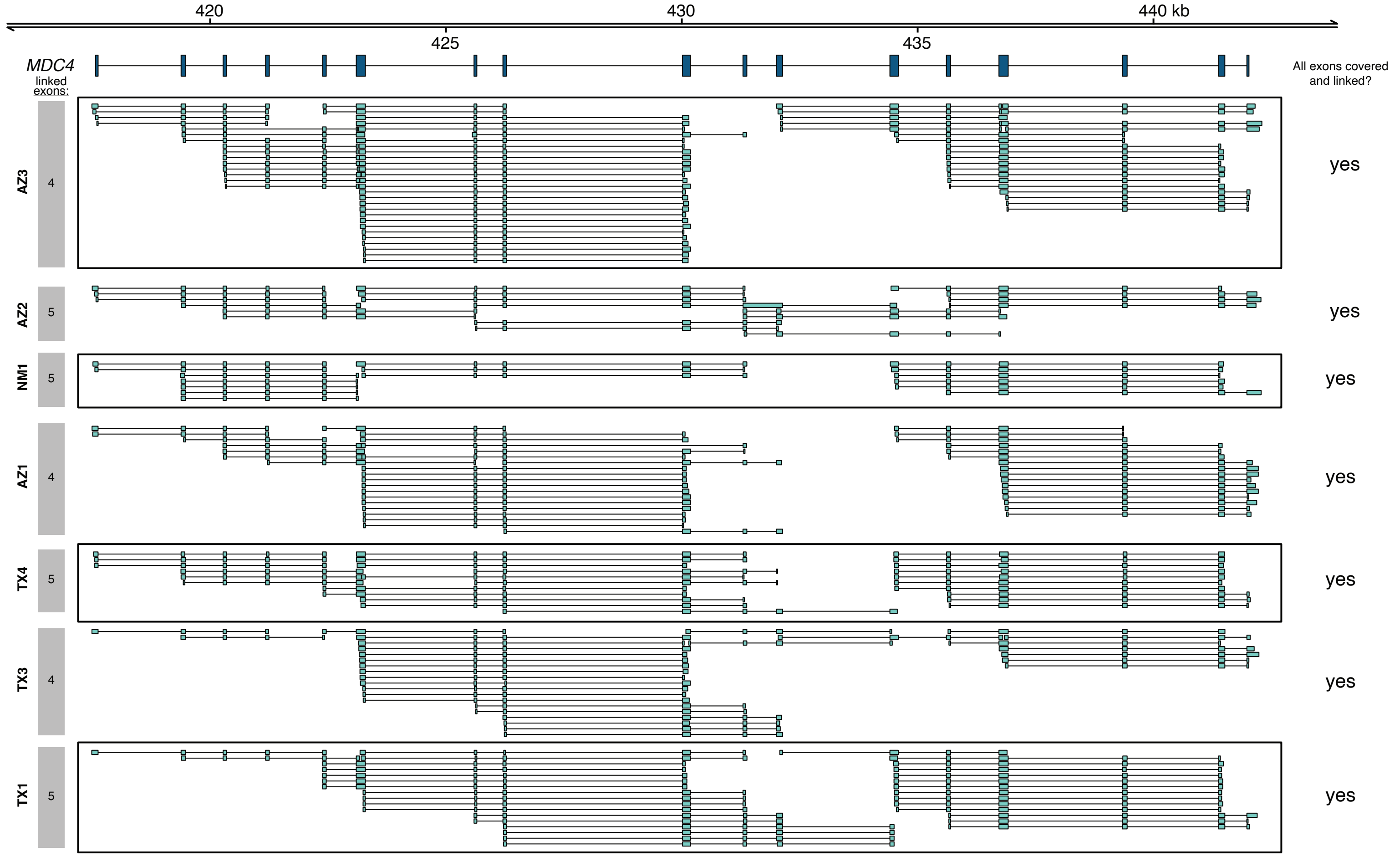

B. Full-length sequences aligning to the *MDC4* gene.

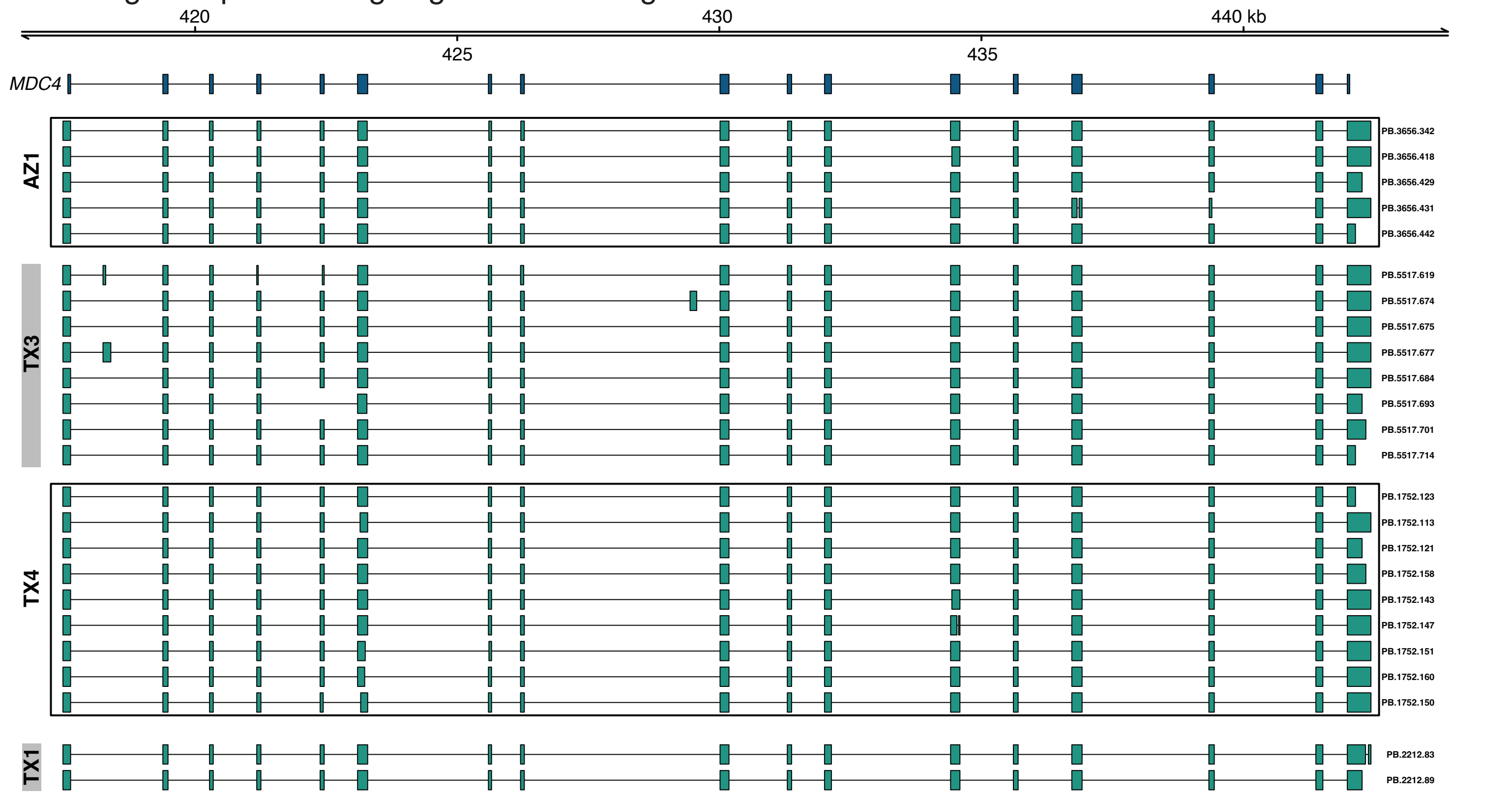

Supplement: S12 Fig — (A) MDC4 assembled transcripts from all specimens tile across the complete gene and link all exons. (B) Full-length MDC4 isoforms identified using single molecule sequencing. (PDF) [file pone.0319316.s009.pdf]
