# Supplementary material for: Loss of a major venom toxin gene in a Western Diamondback rattlesnake population
Source: PLoS One. 2025 Jul 3;20(7):e0319316. doi: 10.1371/journal.pone.0319316 (PMC12225875; doi:10.1371/journal.pone.0319316)

Supplementary Figure S13 Isoforms of *MDC4* are detected but often expression is limited to a subset of individual specimens

A

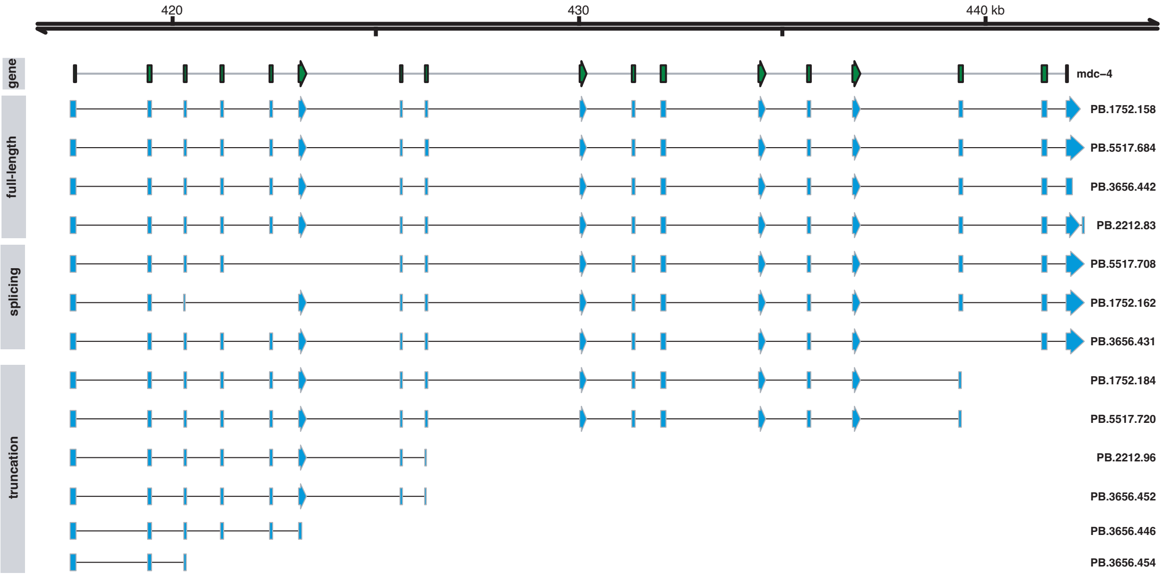

B

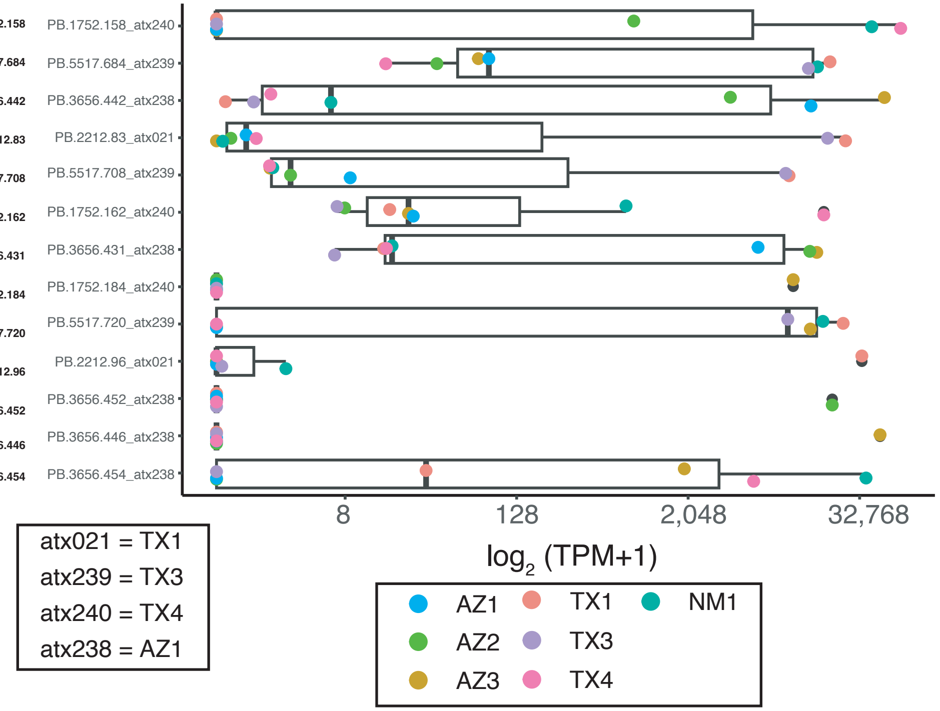

Supplement: S13 Fig — (A) Isoform sequencing using Pacific Biosciences technology (IsoSeq) from four specimens (TX1, TX3, TX4, AZ1) followed by quantification of isoform abundance using short reads from all specimens identified isoforms from several general classes (grey boxes on left showing full-length, splicing, truncation) that align to the genomic region of MDC4 (blue rectangles or arrows show regions of isoform sequence that align, green rectangles in top row show location of annotated MDC4 exons. (B) Flipped box plots with the isoform-level counts (Transcripts Per Million) for seven specimens. Expression is limited or not detected in most specimens for most isoforms. For the three putative full-length isoforms (PB.1752.158_atx240 (TX4), PB.5518.684_atx239 (TX3), PB-3656.442_atx238 (AZ1)) hypothetical translations of only one (PB.1752.158_atx240 (TX4)) yields a full-length protein with the other two isoforms containing nucleotide substitutions that yield a hypothetical truncated protein. (PDF) [file pone.0319316.s010.pdf]
