# Supplementary material for: Loss of a major venom toxin gene in a Western Diamondback rattlesnake population
Source: PLoS One. 2025 Jul 3;20(7):e0319316. doi: 10.1371/journal.pone.0319316 (PMC12225875; doi:10.1371/journal.pone.0319316)

Supplementary Figure S14

Signals from “MPO1 doublet” and lower bands are reduced with excess MPO1 peptide

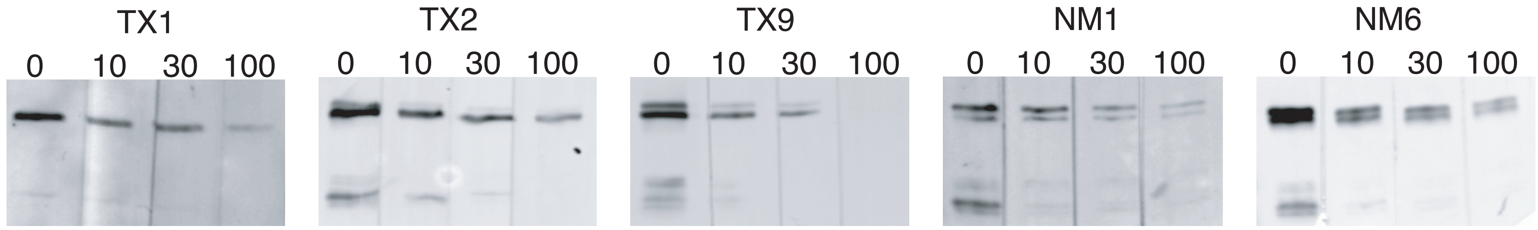

Supplement: S14 Fig — Signal from “MPO1 doublet” and lower bands are reduced with excess MPO1 peptide. Initial characterization of the polyclonal MPO1 antibody and screening of individual C. atrox venoms revealed a doublet at ~25 k Da and a lower molecular weight band. These bands may represent non-specific binding activity of the antibody, recognition of post-translationally modified MPO1, an MPO1 allele (doublet) and/or proteolytic processing of MPO1 (lower band). To distinguish between these possibilities, we synthesized the MPO1 peptide antigen and mixed with the polyclonal MPO1 antibody (10, 30, 100 molar excess peptide). These MPO1- antibody-peptide mixtures were used to probe membrane strips from individual venoms (TX1, TX2, TX9, NM1, NM6). (E) The band intensities of the doublet and lower molecular weight decreased with increasing peptide supporting the possibility that the polyclonal antibody is detecting non-canonical MPO1 products (relatively fast and slow migrating polypeptides) that may represent post-translationally modified or proteolytically processed forms of MPO1. (PDF) [file pone.0319316.s011.pdf]
