# Supplementary material for: Loss of a major venom toxin gene in a Western Diamondback rattlesnake population
Source: PLoS One. 2025 Jul 3;20(7):e0319316. doi: 10.1371/journal.pone.0319316 (PMC12225875; doi:10.1371/journal.pone.0319316)

Supplementary Figure S18

A. Genomic read coverage at *MPO1* and *MDC4* for all specimens

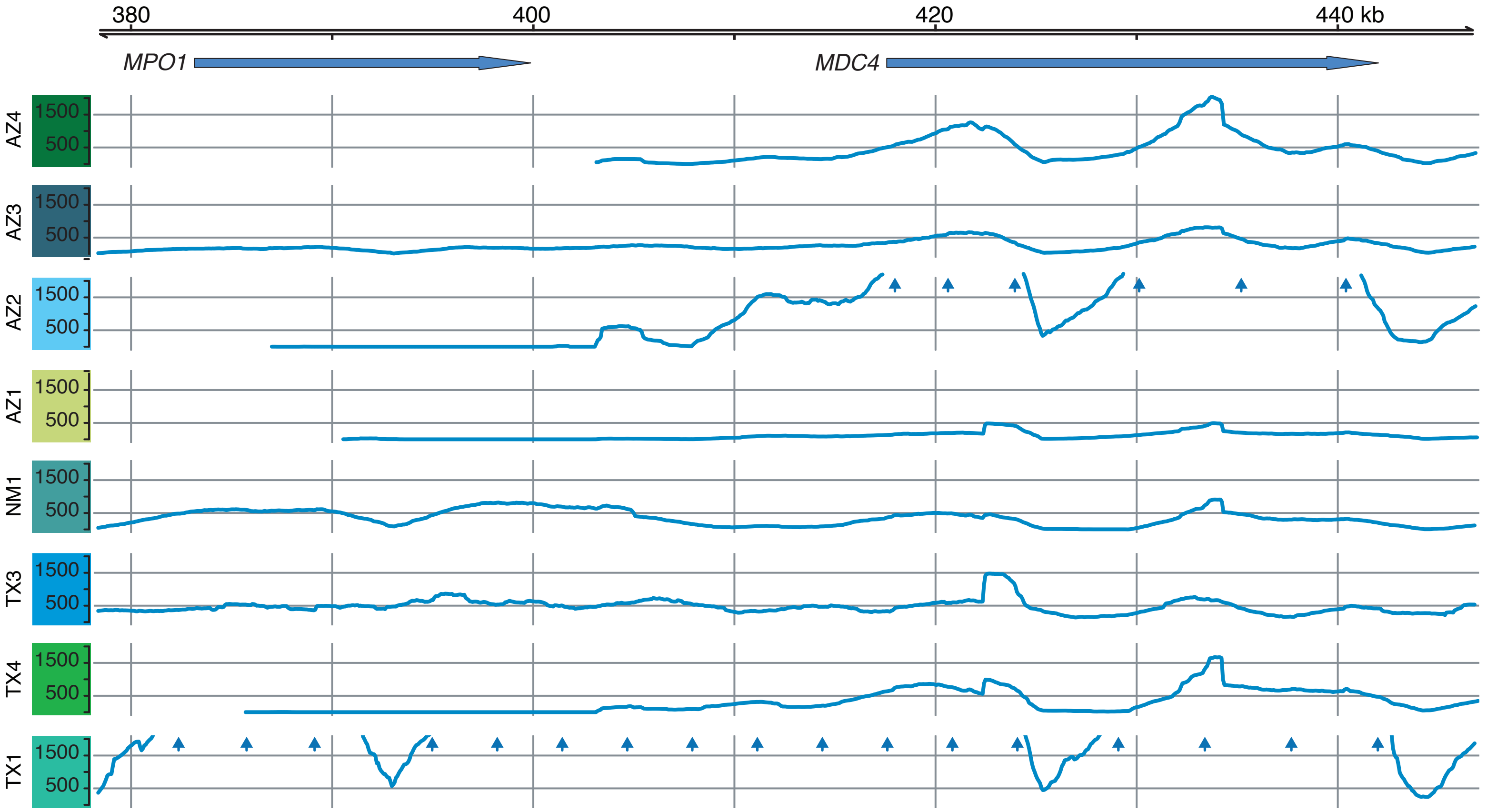

B. Genomic read coverage for two specimens with high coverage

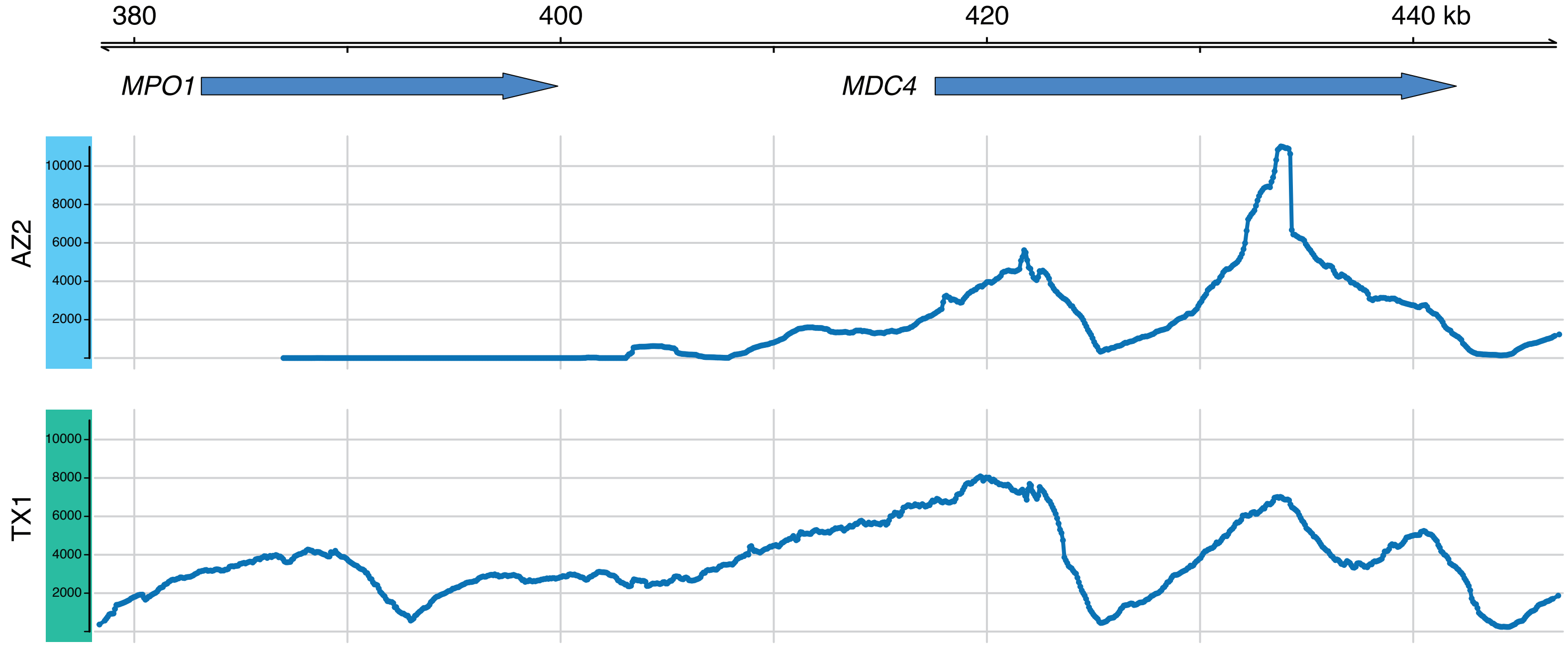

Supplement: S18 Fig — (A) Aligned read coverage at the MPO1 and MDC4 loci for four western (AZ1–4) and eastern (TX1–3 and NM1) specimens. Four specimens (AZ1, AZ2, AZ4, TX4) have zero or low coverage at the MPO1 gene but the adjacent MDC4 gene has similar coverage relative to the other specimens. Prior work generated a reference genome using specimen TX1 and targeted genomic sequencing of TX1 yielded high cover across the total MPO1 - MDC4 region (upward arrows indicate coverage exceeds the y-axis). (B) High genomic read coverage at the MPO1-MDC4 region for two specimens (TX1 and AZ2) with very high coverage. (PDF) [file pone.0319316.s015.pdf]
