# Supplementary material for: Loss of a major venom toxin gene in a Western Diamondback rattlesnake population
Source: PLoS One. 2025 Jul 3;20(7):e0319316. doi: 10.1371/journal.pone.0319316 (PMC12225875; doi:10.1371/journal.pone.0319316)

# Supplementary Figure S20 *C.atrox* assembled contig alignments at *MPO1* and *MDC4* genes

A.

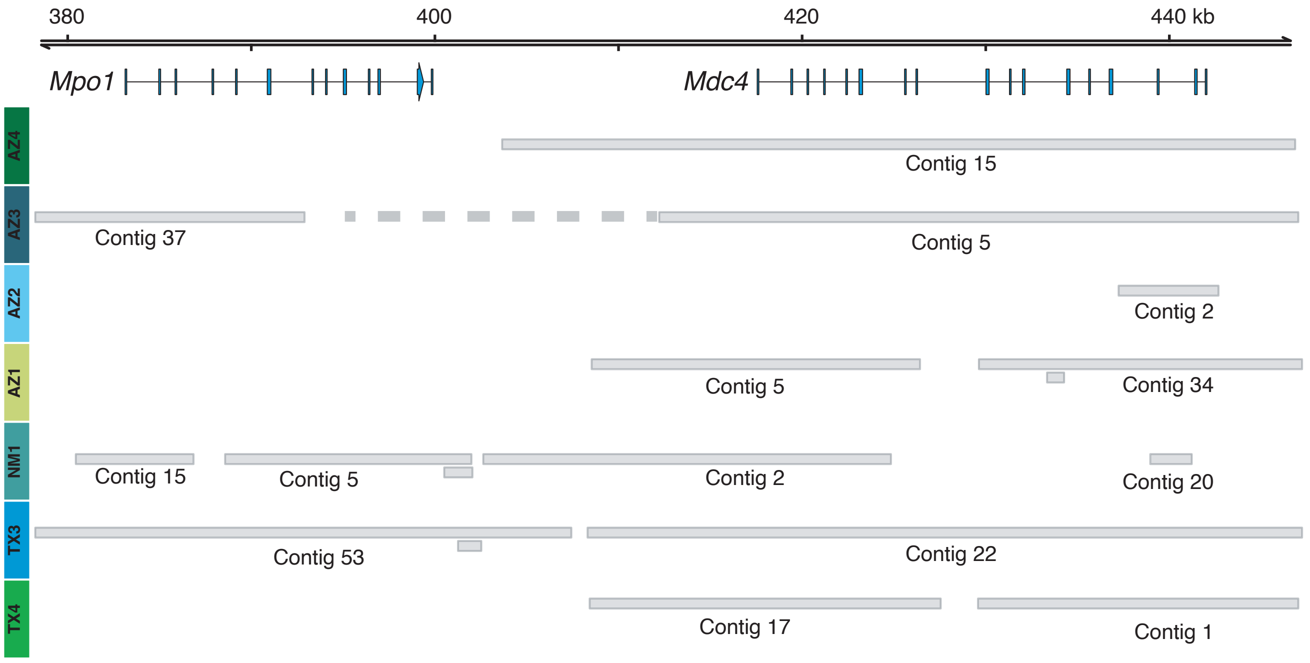


## B. Summary of *MPO1* annotation

| Specimen | Contig | AA sequence % similarity Metalloproteinase domain (MPO1/MAD5a) 7 | 8 | 9 | 10 | 11 | 12 | 14 | alignment summary | phylogenetic clustering with MPO1 |
|---|---|---|---|---|---|---|---|---|---|---|
| AZ3 | 37 (Exons 1-6 AA seqs match MPO1) | | | | | | | | recombination between MPO1 & MAD5a | No (MAD5a) |
| AZ3 | 5 (dash) | 99/**100** | 71/**100** | 73/**100** | 76/**100** | 63/63 | **98**/52 | **100**/- | | |
| NM1 | 5 | 79/**100** | 71/**96** | **88**/82 | **50****/43 | **91**+/60 | **94**#/38 | 100/- | MPO1 | Yes |
| TX3 | 53 | **84***/68 | **88**/79 | **100**/73 | **100**/76 | **67**^/49 | **98**/52 | **100**/- | MPO1 | Yes |

+ = 100 % identical to atrolysin C sequence

# = all 3 substitutions are identical to atrolysin C sequence

** = translation frame 1 has higher identity sequence to MPO1 reference compared to MADa

* = translation frame 2 has higher identity sequence to MPO1 reference compared to MADa

^ = first 28 amino acids 100 % identical to reference MPO1 sequence then a nucleotide insertion changes the final 16 amino acids

## C. Nucleotide sequence exon 10 from *MPO1*

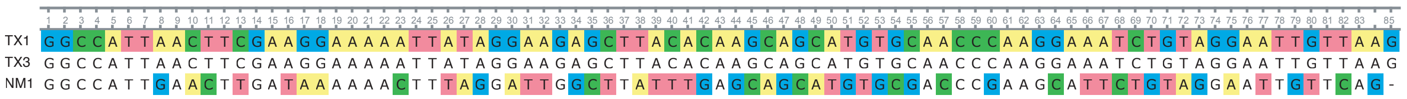


## D. Hypothetical translation of exon 10 from *MPO1*

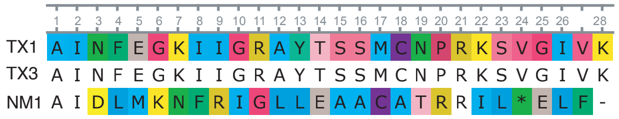


## E. Nucleotide sequence exon 10 from *MPO1* accounting for putative indels in alignment of NM1

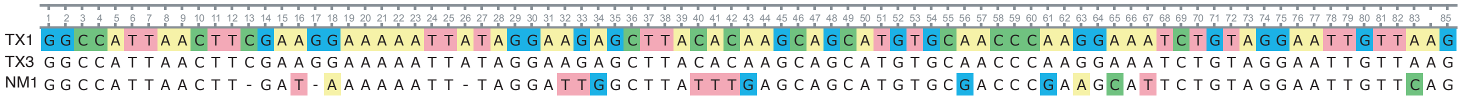


## F. Accounting for indels in hypothetical translation of exon 10 from *MPO1*

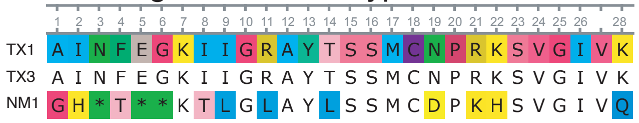

Supplement: S20 Fig — (A) Contigs that align to the MPO1 region were annotated to identify exons. For specimen AZ3, contig 37 aligns to the 5’ region of MPO1 and contains intact exons 1–6 which encode the signal peptide and pro-domain. Contig 5 aligns to MDC4 and extends towards MPO1 but aligns poorly (dashed region). (B) Annotation of this sequence (AZ3, contig 5) revealed metalloproteinase exons 7–12, plus 14. Amino acid percent similarity of exons 7–10 suggests these are MAD5a exons but exons 12 and 14 are nearly identical (98 and 100%, respectively) to MPO1 thus this may be a chimeric gene fusion created during a recombination event. In the reference assembly MAD5a does not contain an exon 14 and is not adjacent to MDC4. Percent identity of protein sequences identified from the annotation of contig 53 in TX3 supports the identification of MPO1 however, a nucleotide insertion in exon 11 results in a hypothetical protein sequence that differs from the reference MPO1. With respect to NM1, annotation exon 10 (contig 5) reveals indels that are predicted to result in translation stop, while exons 11 and 12 are 100% identical (see call outs + and #) to MPO-C (Atrolysin-C) suggesting the identification of a mutated allele of MPO on the assembled contig. (C – F) Alignments of NM1 exon 10 nucleotides (C and E) or amino acids from a hypothetical translation (D and F) show the disruption of coding frame (C, D) that is partially corrected when indels are accounted for (E, F). Compare C and E alignments at positions 13, 17 and 26. Hypothetical translation of the exon 10 without accounting for indels yields a sequence highly divergent from the reference MPO1 and contains a stop codon (D, position 23). (F) Accounting for the indels in a hypothetical translation shows the amino acid sequence similarity between NM1 and TX1 exon 10 sequences at the 3’ segment. (PDF) [file pone.0319316.s017.pdf]
